# Structural assembly of the nucleic-acid-binding Thp3–Csn12–Sem1 complex functioning in mRNA splicing

**DOI:** 10.1093/nar/gkac634

**Published:** 2022-07-29

**Authors:** Zhiling Kuang, Jiyuan Ke, Jiong Hong, Zhongliang Zhu, Liwen Niu

**Affiliations:** Hefei National Laboratory for Physical Sciences at the Microscale, Division of Molecular and Cellular Biophysics, University of Science and Technology of China, Hefei, Anhui 230026, China; School of Life Sciences, University of Science and Technology of China, Hefei, Anhui 230026, China; Institute of Health and Medicine, Hefei Comprehensive National Science Center, Northwest corner of Susong Rd & Guanhai Rd, Hefei, Anhui 230601, China; School of Life Sciences, University of Science and Technology of China, Hefei, Anhui 230026, China; Hefei National Laboratory for Physical Sciences at the Microscale, Division of Molecular and Cellular Biophysics, University of Science and Technology of China, Hefei, Anhui 230026, China; School of Life Sciences, University of Science and Technology of China, Hefei, Anhui 230026, China; Hefei National Laboratory for Physical Sciences at the Microscale, Division of Molecular and Cellular Biophysics, University of Science and Technology of China, Hefei, Anhui 230026, China; School of Life Sciences, University of Science and Technology of China, Hefei, Anhui 230026, China

## Abstract

PCI domain proteins play important roles in post-transcriptional gene regulation. In the TREX-2 complex, PCI domain-containing Sac3 and Thp1 proteins and accessory Sem1 protein form a ternary complex required for mRNA nuclear export. In contrast, structurally related Thp3–Csn12–Sem1 complex mediates pre-mRNA splicing. In this study, we determined the structure of yeast Thp3^186–470^–Csn12–Sem1 ternary complex at 2.9 Å resolution. Both Thp3 and Csn12 structures have a typical PCI structural fold, characterized by a stack of α-helices capped by a C-terminal winged-helix (WH) domain. The overall structure of Thp3^186–470^–Csn12–Sem1 complex has an inverted V-shape with Thp3 and Csn12 forming the two sides. A fishhook-shaped Sem1 makes extensive contacts on Csn12 to stabilize its conformation. The overall structure of Thp3^186–470^–Csn12–Sem1 complex resembles the previously reported Sac3–Thp1–Sem1 complex, but also has significant structural differences. The C-terminal WH domains of Thp3 and Csn12 form a continuous surface to bind different forms of nucleic acids with micromolar affinity. Mutation of the basic residues in the WH domains of Thp3 and Csn12 affects nucleic acid binding *in vitro* and mRNA splicing *in vivo*. The Thp3–Csn12–Sem1 structure provides a foundation for further exploring the structural elements required for its specific recruitment to spliceosome for pre-mRNA splicing.

## INTRODUCTION

In eukaryotic cells, transcription and transcription-associated processes such as pre-mRNA processing and mature mRNA nuclear export are highly coordinated to ensure accuracy and efficiency of gene expression (1). A large number of macromolecular machineries, protein complexes are involved through directly or indirectly associated with RNA and RNA polymerase II. Among them, the highly conserved TREX and TREX-2 complexes are central for mRNA packaging, coupling transcription and mRNA processing with mRNA nuclear export and contributing to transcription-associated genomic stability (1–4). The TREX complex is formed by the association of Yra1 and Sub2 with the THO complex, contributing to the recruitment and loading of the yeast mRNA export receptor Mex67-Mtr2 onto pre-mRNA (5–8). The yeast TREX-2 complex consists of Sac3, Thp1, Sus1, Cdc31 and Sem1, which could mediate association of actively transcribing genes with the nuclear pore complexes (NPCs) to facilitate efficient entry of mRNA nucleoprotein particles (mRNPs) into the NPC channel (9–11).

Functionally associated with TREX and TREX-2, a less well-characterized Thp3–Csn12–Sem1 complex is recruited to the actively transcribed genes functioning in transcription elongation and mRNA splicing (12,13). Csn12 was initially described as a subunit of *Saccharomyces cerevisiae* COP9 signalosome complex (CSN) (14). CSN cleaves Rub1 (Nedd8 in higher eukaryotes) from the Cullin subunit of Cullin-RING ubiquitin ligases (CRLs), and negatively regulates CRL activity (15,16). However, deletion of Csn12 failed to accumulate the neddylated Cdc53 as observed for other subunits of CSN (16), suggesting the distinctive role of Csn12 beyond regulating CRL activity. Only Csn12, but not other subunits of CSN, could physically interact with a number of proteins (SMB1, SMX2 and SMX3) involved in mRNA splicing (17). Mass spectrometry-based protein interaction analysis showed that Thp3 (also called Ypr045c) could co-enrich with tandem affinity purification (TAP)-tagged Csn12 in budding yeast (12). It was then confirmed that Csn12 and Thp3 could interact specifically to form a complex independent of the rest subunits of CSN (12). The Thp3–Csn12 complex was functionally linked to spliceosome. Deletion of either Csn12 or Thp3 had a splicing defect, causing accumulation of intron-containing pre-mRNA, which was similar to *isy1Δ* strain and several other splicing factor mutants (13).

Yeast Sem1 (its human homolog DSS1) is a small intrinsically disordered protein that is evolutionally conserved and involved in assembly of many different protein complexes functioning in diverse biological processes: i.e. as a 26S proteasome subunit important for proteasome assembly (18), and ubiquitin recognition (19), in complex with the BRCA2 to interact with the single-strand DNA-binding protein RPA involved in DNA repair (20,21), and association with the Integrator complex for the small nuclear RNA (snRNA) processing (22), in complex with Sac3-Thp1 of the TREX-2 complex functioning in mRNA export and transcription elongation (23). The versatile small Sem1 protein also serves as an accessory protein to associate with Thp3–Csn12 complex (24). Sem1 co-purified with Csn12 and Thp3, indicating an important role of Sem1 in the Thp3–Csn12 complex assembly although loss of Sem1 did not significantly impair function of the Thp3–Csn12 complex in mRNA splicing (4,13,24).

It is intriguing to understand how Thp3–Csn12–Sem1 complex is associated with spliceosome for mRNA splicing. Additionally, how does Thp3–Csn12–Sem1 complex coordinate with TREX-2 complex to facilitate mRNA splicing and mRNA nuclear export in an orderly manner? Here, we set out to understand the structural and biochemical basis of Thp3–Csn12–Sem1 complex assembly. Bioinformatics and structural analyses revealed that both Csn12 and Thp3 belong to PCI family of proteins, which exhibit similar sequence region characterized by a superhelical (HD) domain, followed by a conserved α/β winged helix (WH) domain (23). Csn12 harboring a typical PCI domain with the HD region was further designated as PCI associated module (PAM) fold (24). Its relatively conserved sequence property was only found among a subset of special PCI domain-bearing proteins such as Thp1 (23). Thp3 was assigned to SAC3-GANP subtype originally identified in the Sac3 subunit of TREX-2 and its human orthologue GANP (23,25,26). Hence, Thp3–Csn12–Sem1 complex appears to be a structural paralogue of Sac3–Thp1–Sem1 complex with a distinctive function in mRNA splicing (23).

In the current study, we reported the first biochemical and structural basis of Thp3–Csn12–Sem1 complex. We found that Csn12 was only soluble when co-expressed with Sem1, GST-tagged Thp3 or both of them. Our *in vitro* binding assay showed that Csn12 formed a stable complex with Thp3 and Sem1, which could be readily purified to homogeneity from *E. coli*. To understand the complex assembly, we determined the crystal structure of the truncated Thp3^186–470^ in complex with Csn12 and Sem1 (Thp3^186–470^–Csn12–Sem1 ternary complex) at 2.9 Å. The structure revealed an overall similar structural architecture with Sac3–Thp1–Sem1 complex (23). Our analysis further highlighted significant structural differences between them, which may account for their distinctive functions in mRNA splicing versus mRNA nuclear export. In the Sac3–Thp1–Sem1 complex, the juxtaposition of WH domains of Sac3 and Thp1 generated a platform for binding nucleic acids (23). Based on the structural similarity, we examined the nucleic acid binding activity of Thp3–Csn12–Sem1 complex with fluorescence polarization (FP) assay. Indeed, Thp3–Csn12–Sem1 complex could bind different forms of nucleic acids. A nucleic acid binding surface could be located on the complex structure and structure-based mutagenesis of the positively charged residues on this surface affected DNA/RNA binding *in vitro* and mRNA splicing *in vivo*. Together, the detailed structural and complex assembly information will help understand the in-depth molecular basis of mRNA splicing mediated by the Thp3–Csn12–Sem1 ternary complex.

## MATERIALS AND METHODS

### Yeast strains, plasmids and growth conditions

*S. cerevisiae* strain BY4742 (MATα; his3Δ1; leu2Δ0; lys2Δ0; ura3Δ0) and plasmids (pUG6 and pEUGAP) were used in this study. Yeast cells were grown at 30°C. Yeast culture media included YPDA (10 g/l yeast Extract, 20 g/l peptone and 20 g/l dextrose and 120 mg/l adenine hemisulfate), minimal medium (6.7 g/l yeast nitrogen base, 20 g/l dextrose and 1.92 g/l yeast synthetic dropout medium supplements without uracil), or 5-FOA medium (minimal medium supplemented with 0.02 mg/ml uracil and 1 mg/ml of 5-fluoro-orotic acid).

### Cloning, small scale protein expression and pull down assay

The DNA fragments corresponding to Csn12 and Sem1 were PCR-amplified utilizing gDNA from *S. cerevisiae* and cloned into the first and second MCS of pETDuet-1 (Novagen) using NcoI and NotI, NdeI and XhoI restriction sites respectively. The full-length or truncated cDNA fragment (residues 186–470) of Thp3 was inserted into a modified pET28a plasmid (Novagen) that has been cloned with an N-terminal 8×His-glutathione S-transferase (GST) tandem tag followed by a TEV protease cleavage site. All the site-directed mutagenesis were created with a PCR-based method and verified by DNA sequencing. For Csn12 single expression, *E. coli* Rosetta (DE3) cells were transformed with pET-22b (Novagen) cloned with the full-length Csn12 fused with a C-terminal 6×His tag. For Thp3 single expression, *E. coli* Rosetta (DE3) cells were transformed with a modified pET28a plasmid (Novagen) cloned with the full-length wild-type or mutant Thp3 fused in frame with an N-terminal 8×His-GST tandem tag (His8-GST-Thp3). The Csn12 protein alone formed inclusion bodies in *E. coli*. 8×His-GST-Thp3 was expressed as a soluble protein in *E. coli*, but it formed multimer and was unstable in solution. We were unable to obtain sufficient quantities of each individual protein for biochemical studies. Therefore we turned our attention to coexpress these proteins and use pull-down assay to analyze complex formation. For Csn12 and Sem1 co-expression, cells were transformed with pETDuet-1 (Novagen) cloned with 6×His tagged full-length wild-type Csn12 and untagged Sem1 at the first and second MCS, respectively. For Thp3 and Sem1 co-expression, cells were co-transformed with a modified pET28a plasmid cloned with the His8-GST-Thp3 and a pETDuet-1 plasmid cloned with the untagged Sem1. For Thp3 and Csn12 co-expression, cells were co-transformed with a modified pET28a plasmid cloned with the His8–GST–Thp3 and a pET-22b plasmid cloned with an untagged Csn12. For Thp3, Csn12 and Sem1 co-expression, cells were transformed with a modified pET28a plasmid cloned with the His8-GST-Thp3, and a pETDuet-1 plasmid cloned with untagged Csn12 and untagged Sem1 at the first and second cloning sites.

For His pull down assay, cleared cell lysates were incubated with Ni affinity resins pre-equilibrated with buffer A (50 mM Tris pH 8.0, 500 mM NaCl). The protein bound beads were sequentially washed with buffer A supplemented with 20 and 50 mM imidazole, followed by eluting the protein complex with buffer A containing 300 mM imidazole. The eluted protein complexes were evaluated by SDS-PAGE.

### Large scale protein expression and purification

The corresponding transformants were grown in LB media to an OD_600_ of ∼0.6 at 37°C, followed by inducing the protein expression with 0.5 mM isopropyl β-d-thiogalactoside (IPTG) at 16°C for ∼20 h. To express the selenomethionine (SeMet)-substituted protein complex, clones were inoculated in M9 minimum medium at a ratio of 1:100 until the cell density reached an OD_600_ of ∼1.0. Cells were grown for additional 20 min at 25°C in the cell culture media supplemented with 100 mg/l of lysine, phenylalanine, threonine, isoleucine, leucine and valine. Following that, 70 mg/l of selenomethionine was added to the cell culture and induced with 0.5 mM IPTG at 16°C for ∼30 h.

Cells expressing the full-length or truncated Thp3 (residues 186–470) in complex with full length Csn12 and Sem1 proteins were suspended in buffer B (50 mM Tris pH 8.7, 500 mM NaCl), followed by sonication on ice and centrifugation at 12 000 rpm for 30 min at 4°C to remove cell debris. Then the ternary complex was purified to homogeneity utilizing consecutive chromatographic purifications such as immobilized Ni-NTA affinity, gel filtration and cation exchange chromatography. Briefly, clarified supernatant was loaded onto a 5 ml Ni-NTA column (Sigma Aldrich, USA) pre-equilibrated with buffer B. After sequential washes with ten column volumes of buffer B supplemented with 20 mM and 50 mM imidazole, the bound protein complex was eluted with buffer B supplemented with 300 mM imidazole. The eluted protein complex was further purified with HiLoad 16/60 Superdex 200 (GE Healthcare, USA) pre-equilibrated with buffer B. Fractions containing the ternary complex were pooled and incubated with TEV protease at a molar ratio of 1:20 at 16°C overnight to remove the 8×His-GST tag from Thp3 protein. Cleaved protein complex was further purified with SP Sepharose Fast Flow column (HiTrap™ FF, GE Healthcare, USA) and eluted with a linear gradient of 0.2–1.0 M NaCl. The purity of the protein complex was verified with SDS-PAGE. Purified protein was concentrated to 20–30 mg/ml in buffer C containing 50 mM Tris (pH 8.7), 200 mM NaCl, 5 mM DTT and 1 mM EDTA using a Millipore concentrator (Amicon, USA) and stored at −80°C. Please note that the C-terminal His6-tag on Csn12 was removed for the purified ternary complex. Similar protocol was also applied for purification of the SeMet-labeled protein complex.

### Crystallization and data collection

The protein complexes were diluted to 10 mg/ml, followed by mixing an equal volume of protein and reservoir solution at 16°C utilizing sitting-drop vapor diffusion method. We initially tried to crystallize the full length Thp3–Csn12–Sem1 ternary complex. However, we were unable to obtain the good quality diffraction crystal which might be possibly due to the flexible regions of Thp3 and Csn12 subunits. Our secondary structure prediction and multiple sequence alignment results revealed that Thp3 has a divergent, flexible N-terminal region followed by highly conserved middle and C-terminal regions (Supplementary Figure S1), whereas full length Csn12 presented regular secondary structures. Hence, we devoted our efforts to crystallize the truncated Thp3 (residues 138–470) in complex with full-length of Csn12 and Sem1. However, crystallization of this ternary complex also failed. Subsequently we used spontaneous degradation-connected mass spectrometric analysis to identify any unstable region in Thp3 and found a stable degradation product (product 1) of Thp3 corresponding to residues 186–470 (Supplementary Figure S2). Hence, we finally focused on co-crystallization of Thp3^186–470^ with Csn12 and Sem1.

For crystallization, the ternary complex of Thp3 (186–470), Csn12 and Sem1 was diluted with buffer C to a final concentration of 10 mg/ml. The crystals were grown using the sitting-drop vapor diffusion method by mixing 1 μl of the ternary complex solution with 1 μl of reservoir solution, and the well contained 100 μl of the reservoir solution. Crystallization screens were set up at 16°C using Crystal Screen, Crystal Screen 2, PEG/Ion 1 Screen, PEG/Ion 2 Screen, Salt Rx and Index from Hampton Research and Proplex from Molecular Dimensions. Good quality hexahedral crystal was grown in the solution containing 12% PEG3350, 100 mM sodium malonate (pH 5.0) and 3% methanol. Similarly, SeMet-labeled crystals for phasing were also obtained under the same condition. For data collection, crystals were cryoprotected in the above-mentioned reservoir solution supplemented with 18% glycerol, followed by flash-freezing into liquid nitrogen. The single wavelength diffraction data set for wild-type crystal and single wavelength anomalous dispersion (SAD) diffraction data set for SeMet-substituted crystal were collected on the beam line 18U1 at Shanghai Synchrotron Radiation Facility (SSRF) at a wavelength of 0.9785 Å at 100 K.

### Spontaneous protein degradation analysis

Since Thp3^138–470^–Csn12–Sem1 complex was not stable during crystallization, spontaneous degradation analysis of the complex was performed to find the suitable domain boundary for crystallization. Briefly, 2 mg/ml of purified protein complex in the reaction buffer (50 mM Tris (pH 8.7), 500 mM NaCl, 5 mM DTT, 1 mM EDTA) was incubated at 4°C and 16°C for several days. Then 10 μl aliquot of the protein samples after degradation was taken at 0, 1, 3, 5 and 7 days and analyzed by SDS-PAGE with Coomassie blue staining. The protein bands corresponding to the two stable degradation products of Thp3^138–470^ were excised and analyzed with mass spectrometry.

### Structure determination and refinement

Crystal diffraction data sets were processed with HKL2000 program (27). The initial experimental phases were determined by SAD data set of SeMet-substituted crystal utilizing CRANK2 program (28) from CCP4i suite. Further automatic model building and extension was carried out with BUCCANEER (29) followed by iterative rounds of manual model building and refinement using Coot (30) and Refmac5 (31), respectively. A Translation-Libration-Screw-rotation (TLS) refinement (32) in Phenix was performed at the last stage and the final SeMet model was refined to convergence with the corresponding *R*_work_/*R*_free_ of 21.20%/25.67%. Structure stereochemistry was validated with PROCHECK (33) and MolProbity (34) programs. The final SeMet model has good geometry with 98.77% and 1.23% of residues in the favored and allowed regions in the Ramachandran plot. The refined SeMet structure model was used as a search model to solve the native structure at 2.9 Å by molecular replacement (MR). The final native structure was refined at the resolution of 2.9 Å with the corresponding *R*_work_/*R*_free_ of 22.20%/25.10% with good geometry (98.77%, 1.23% and 0.00% of the residues in the favored, allowed and disallowed regions, respectively). Statistics for data collection and refinement were summarized in Table 1. Figures presenting the structural model were prepared with PyMol (35).

**Table 1.** X-ray data collection and refinement statistics

Data collection	Thp3(186–470) + Csn12 + Sem1 SeMet	Thp3(186–470) + Csn12 + Sem1 Native
Space group	*P*3_2_21	*P*3_2_21
Cell dimensions		
*a, b, c* (Å)	116.3, 116.3, 127.3	115.6, 115.6, 126.8
α, β, γ (°)	90, 90, 120	90, 90, 120
*R*_merge_ (%)	14.70 (89.5)	11.50 (106.0)
*Rpim* (%)	3.70 (21.7)	2.70 (24.1)
CC_1/2_ (%)	100.00 (89.70)	99.50(88.80)
*I*/σ*I*	23.44 (2.62)	29.77 (2.36)
Completeness (%)	100.00 (97.30)	99.90 (97.00)
Redundancy	16.60 (17.60)	19.30 (20.10)
**Refinement**		
Resolution (Å)	33.58–2.85 (2.98–2.85)	32.30–2.90 (3.03–2.90)
No. reflections	23 630 (2929)	22 146 (2748)
*R*_work_*/R*_free_	21.20/25.67	22.20/25.10
*B*-factors(Å^2^)	87.95	96.47
R.m.s.deviations		
Bond lengths (Å)	0.005	0.006
Bond angles (°)	0.823	0.878
Ramachandran plot (%) (favored/ allowed/outliers)	98.77/1.23/0.00	98.77/1.23/0.00
MolProbity score (percentile)	1.60 (100)	1.64 (100)

### Fluorescence polarization assay

To measure the nucleic acid-binding activity, fluorescence polarization assay was performed in the black 96-well plate at 25°C utilizing a SpectraMax M5 microplate reader system with the fluorescence excitation and emission wavelengths of 485 and 520 nm, respectively. 50 nM of 5′-FAM-labeled nucleic acid probes of different lengths ranging from 15 to 25 nts in 5-nt increment and types (single or double-stranded DNA, single-stranded RNA) were incubated with different concentration of proteins (ranging from 25.6 to 0 μM in a 2-fold serial dilution) in the FP buffer (20 mM Tris–HCl (pH 8.0), 150 mM NaCl, 2 mM MgCl_2_) in a total volume of 200 μl. All samples were incubated at room temperature for 30 min before measurement. The fluorescence polarization signal P in mP units was calculated as the ratio of difference between vertical and horizontal emission intensities to the sum of vertical and horizontal emission intensities. The mean values (± standard deviation, SD) of the experimental data from three independent measurements were plotted and fitted to a single-site binding model according to the following formula:

$$\begin{eqnarray*} && {\rm{F}}{{\rm{P}}_{{\rm{overall}}}}{\rm{ = F}}{{\rm{P}}_{{\rm{min}}}}{\rm{ + A\times}}( {{\rm{K}}_{\rm{D}}}{\rm{ + }}{{\rm{L}}_{\rm{T}}}{\rm{ + }}{{\rm{R}}_{\rm{T}}}{\rm{ - }}( {( {{{\rm{K}}_{\rm{D}}}{\rm{ + }}{{\rm{L}}_{\rm{T}}}{\rm{ + n\times}}{{\rm{R}}_{\rm{T}}}} )} \nonumber \\ && \quad \wedge {\rm{2 - 4\times{{\rm L}_{\rm T}}\times{{\rm R}_{\rm T}} + }} ) \wedge {\rm{0}}{\rm{.5}} ) / {( {{\rm{2 }}\times{{\rm{n }}}\times{{\rm{R}}_{\rm{T}}}} )}. \end{eqnarray*}$$

The Origin 8 software was used to prepare the fitted binding curves and obtain *K*_D_ values, where *n*, *R*_T_ and *L*_T_ are binding ratio, total nucleic acid probe and protein concentrations, respectively. FP_overall_ represents the total polarization value measured, FP_min_ represents the polarization value of protein-free nucleic acid probe, FP_max_ represents the maximum polarization value when the nucleic acid probe binds to protein sample.

### Deletion or mutation of ISY1, CSN12 or THP3 in yeast

PCR-based target gene deletions (CSN12, THP3 and ISY1, an essential pre-mRNA splicing factor) and site-specific mutations in CSN12 or THP3 were carried out with homologous recombination method utilizing primers listed in Supplementary Table S1 as previously described (36). First, gene deletion cassettes of loxP-kanMX-loxP with 90 bp of homology sequence region flanking the open reading frame (ORF) regions in the chromosomal target gene were amplified from pUG6 and transformed into *S. cerevisiae* (BY4742 strain). Colonies were screened on YPDA agar plates containing 200 μg/ml G418 and verified by PCR. Subsequently, URA3 selectable marker gene substitution cassettes were transformed into BY4742-CSN12Δ::kanMX or BY4742-THP3Δ::kanMX for the removal of kanMX marker and transformants were screened on minimal media containing agar plates (Ura−). BY4742-CSN12Δ::URA3 or BY4742-THP3Δ::URA3 was used as a parental strain for constructing site-specific charge reversal mutations of CSN12 or THP3. Site-specific mutations in CSN12 or THP3 were created by PCR-based methods. The amplified CSN12 or THP3 PCR fragments were flanked by 800 bp of sequence immediately upstream and downstream of the ORF regions in the chromosomal target gene. Selection for CSN12 or THP3 mutant by the loss of URA3 marker was performed by a third homologous recombination with long sequence flanking target gene ORF in 5-FOA medium and verified with PCR and Sanger sequencing.

### Total RNAs extraction, endpoint PCR and quantitative PCR assays

Total RNA was purified from wild-type and mutant yeast cultures growing in log phase at 30°C and converted to cDNA following the manufacturer's protocol (TaKaRa). The yeast cells were collected, and cell wall was disrupted by Lyticase (ZOMANBIO). Total RNAs were extracted using a Yeast Total RNA Isolation Kit according to the manual. Single-stranded cDNAs were synthesized from 1 μg of total RNA using a PrimeScript™ RT reagent Kit with gDNA Eraser (TaKaRa). Using an equal amount of cDNAs as templates and intron-specific primers, gene specific pre-mRNA was amplified by the regular polymerase chain reaction (PCR) for 30 cycles using High-Fidelity PrimeSTAR DNA Polymerase (TaKaRa). Similar amplification protocol was also applied for intron-less ALG9 gene which was used as a loading control. The PCR-amplified cDNA products were examined by 2% agarose gel electrophoresis. Using an equal amount of cDNAs, quantitative PCR (qPCR) was performed using TB Green® Premix Ex Taq™ II (TaKaRa) and gene-specific primer pairs (Supplementary Table S2). Fluorescence signals were detected and analyzed by LightCycler 96 system (Roche). Each data-point represents the average values (± SD) from three independent measurements. The relative RNA level was calculated with LightCycler 96 software according to the formula;

$$\begin{eqnarray*} {\rm R} &=& {2\wedge} (-\Delta{\rm Cq}), {\rm where}\, \Delta{\rm Cq} = {{\rm Cq}_{\rm target\text{-}gene}} \nonumber \\ && - {{\rm Cq}_{\rm internal\text{-}contronl\text{-}gene}} \end{eqnarray*}$$

*R*_I_ and *R*_T_ represent pre-mRNA and total mRNA levels, respectively. An intron-less gene ALG9 was used as an internal control.

## RESULTS

### Sem1 stabilizes Csn12 and promotes ternary complex formation with Thp3

Based on the previous research that Csn12, Thp3 and Sem1 could form a complex, we examined expression of individual protein alone and coexpresssion of two or three proteins together utilizing His pull down assay (Supplementary Figure S3). We observed soluble expression of 8×His-GST tagged Thp3 protein alone, and coexpression with Sem1 did not further improve the solubility. In contrast, Csn12 was completely insoluble when expressed alone, however, its coexpresssion with Sem1 or 8×His-GST-Thp3 made it soluble (Supplementary Figure S3). Upon coexpresssion of all the three members, 8×His–GST–Thp3, Csn12 and Sem1 could form soluble ternary complex (Supplementary Figure S3). The 8×His–GST–Thp3 and Csn12 binary complex was not very stable, which formed precipitation when either the GST tag is removed or the complex is at high concentration. Instead, Csn12 could form a stable binary complex with Sem1 or a ternary complex with Sem1 and GST–Thp3. These biochemical results indicated that Sem1 interacts more strongly with Csn12 to stabilize its conformation and Thp3 can further interact with Csn12–Sem1 to form a stable ternary complex.

### Overall structure of Thp3^186–470^–Csn12–Sem1 ternary complex

We initially tried to crystallize the full length complex of Thp3–Csn12–Sem1 without success. Using spontaneous degradation-connected mass spectrometry analysis, we found that Thp3 (138–470) was further degraded into N-terminal short fragment and C-terminal long fragment (residue 186–470) while Csn12 and Sem1 proteins were stable (Supplementary Figure S2). We finally purified a stable complex of truncated Thp3 (186–470) in complex with full length Csn12 and Sem1, which allowed us to grow crystals suitable for structural experiments. These results suggested that N-terminal region of Thp3 is flexible and unstable, which might possibly prevent the formation of well-diffracting crystals. The initial complex structure of SeMet-substituted structure was solved by SAD phasing. The native structure was determined by molecular replacement utilizing the SeMet-substituted structure as a search model. The refined SeMet and native structural models belong to the *P*3_2_21 space group, and contain single copy of Thp3–Csn12–Sem1 ternary complex in the asymmetric unit. The ternary complex in the crystal is a heterotrimer of Thp3, Csn12 and Sem1 with a stoichiometric ratio of 1:1:1, which is consistent with the gel filtration result. The 3D structures of native and SeMet-substituted complexes are almost identical with a root mean square deviation (RMSD) of 0.25 Å for over 739 aligned Cα atoms (Supplementary Figure S4). The native complex covers residues 186–470 of Thp3, residues 1–423 of Csn12 and residues 1–89 of Sem1. The final refined model contains most regions except for some disordered regions that were invisible in the final electron density map such as the C terminal residues 459–470 of Thp3, residues 1–5 and an internal loop corresponding residues 370–378 in Csn12, and residues 1–31 at the N terminus of Sem1.

**Figure 1.**
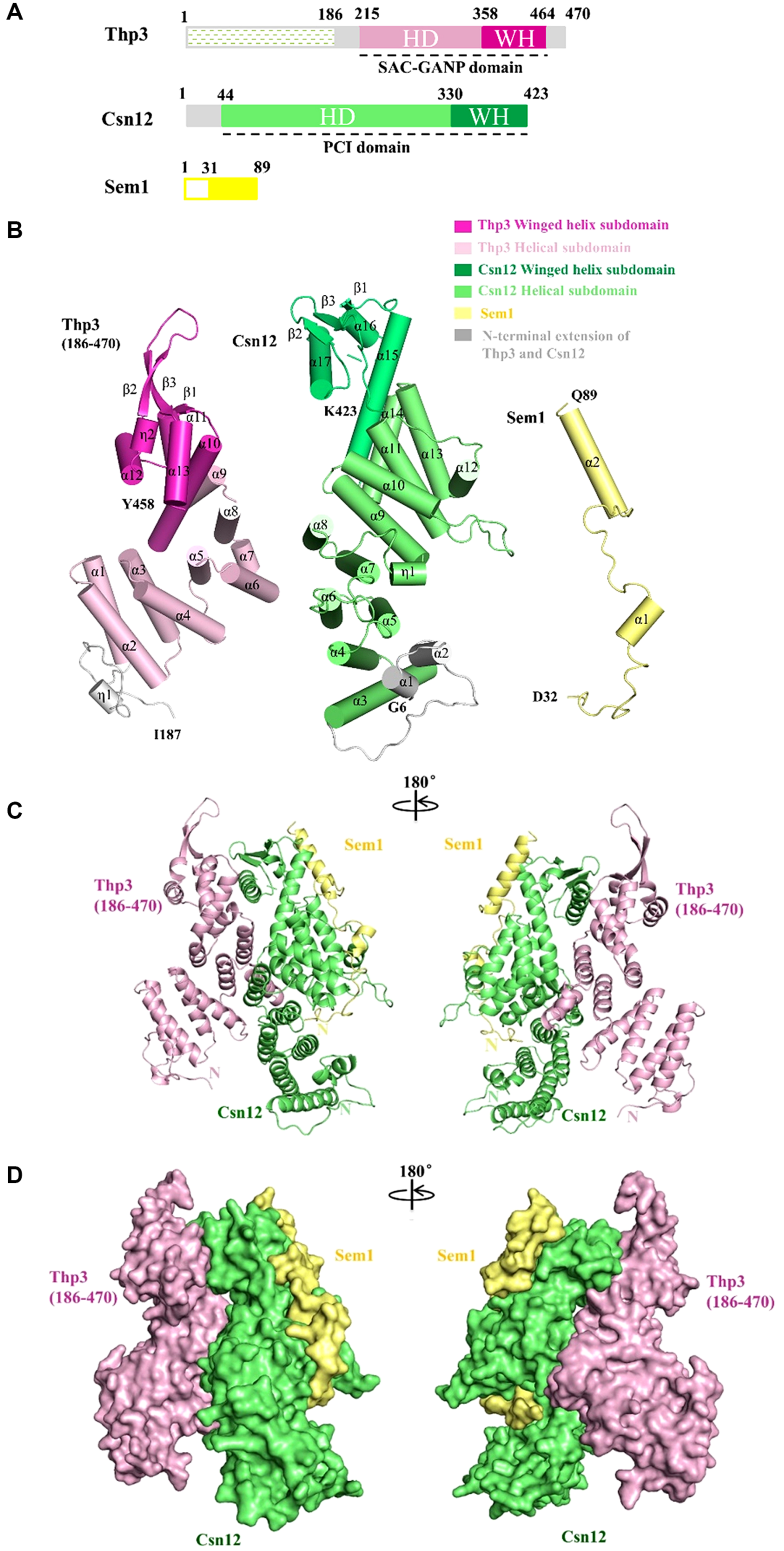
Crystal structure of Thp3–Csn12–Sem1 complex. (**A**) The schematic diagram of Thp3, Csn12 and Sem1 primary sequences with the domain structures and boundaries annotated. The N-terminal region (residues 1–186) of Thp3 that is not in the structure is colored in white with dashed lines. The Thp3 helical domain (HD) and the winged helix domain (WH) are colored in light pink and magenta. The Csn12 HD and WH are colored in light green and green. The N-terminal extensions in Csn12 and Thp3 are colored in gray. Sem1 is colored in pale yellow for residues 32–89 with the N-terminal disordered region (residues 1–31) colored in white. (**B**) Subunit structures of the Thp3–Csn12–Sem1 complex are shown in cartoon representation. The α-helices and β-strands are shown in cylindrical models and arrows. All the secondary structures are labeled. The same color scheme is used as in (A). (**C**) The Thp3–Csn12–Sem1 complex is shown in cartoon representation in two orientations related by a 180°rotation along the y axis. Thp3, Csn12 and Sem1 are colored in light pink, green and yellow respectively. (**D**) Surface representation of Thp3–Csn12–Sem1 complex in the same orientation and color scheme as shown in (C).

Thp3 primary structure contains a flexible N-terminal region, followed by HD and WH domains (Figure 1A). The structure of Thp3 (186–470) consists of thirteen α-helices designated as α1–α13, two 3_10_ helices (η1 and η2), and three β-strands (β1–β3), as illustrated in Figure 1B. The HD is composed of helices α1–α9, which form four pairs of di-helical repeats (α1–α6, α8–α9) packing side by side in a parallel mode to create a superhelical structure with a right-handed twist (Figure 1B). Following that, a compact WH domain is formed by α-helices α10–α12 in association with three β-strands β1–β3 in a topological order of α10–β1–α11–α12–η2–β2–β3. The N-terminal region before α1 forms flexible loop structure packing against α1–α2 of helical domain. The C-terminal region after β3 forms a curved helix α13, which packs against helices α10 and α11 of the WH structure. Notably, the region corresponding to α13 in Thp3 is usually a long loop in many other WH domain-containing proteins.

The primary sequence of Csn12 contains a short N-terminal domain (1–40) followed by a long HD (44–330) and a short WH domain (331–423) (Figure 1A). The full-length Csn12 structure contains seventeen α-helices designated as α1–α17, one 3_10_ helix assigned as η1, and three β-strands labeled as β1–β3 (Figure 1B). The middle HD structure (PAM fold) has an overall right-handed superhelical structure, which consists of six pairs of TPR-like antiparallel di-helical repeats covering helices α3-α14 with connecting loops of variable length (Figure 1B). The N-terminal region corresponding to residues 6–40 adopts two short helices (α1-α2) and a stretched long loop, which stack against helices α3–α5. The C-terminal WH structure consists of helices α15–α17 and strands β1–β3, which arranges in the topological order of α15–β1–α16–α17–β2–β3. The WH folds into a compact α/β structure with β1–β3 forming three-stranded β sheet on the top, supported by helices α16–α17 at the bottom. For Sem1, the N-terminal region (residues 1–31) is disordered in the structure. The N-terminal region corresponding to residues 32–70 forms a stretched loop structure except for residues 51–55, which form a short helix (α1) (Figure 1B). The C-terminal region (residues 71–89) forms a long structured helix (α2). The overall shape of Sem1 looks like a fishhook.

Both Csn12 and Thp3 (186–470) are mostly comprised of helices except that the C-terminal WH domain of each protein contains a 3-stranded antiparallel β-sheet. The overall structure of Thp3^186–470^–Csn12–Sem1 complex has an inverted V-shape with Thp3 and Csn12 forming the left and right sides, respectively (Figure 1C). The helical domains and WH domains roughly form the bottom and top halves of the complex. A fishhook-shaped Sem1 fragment contacts exclusively Csn12. It wraps around the right side surface area of Csn12 to stabilize Csn12 structure extensively (Figure 1C and D). The right-handed superhelical structure in the helical domain at the bottom is connected to WH domain at the top through a long curved helix, i.e. helix α15 in Csn12 and helix α10 in Thp3. A unique feature is the juxtaposed arrangement of Csn12 and Thp3, which connects the two spatially adjacent β-sheets in the WH domains to form a solvent-exposed six-stranded β sheet at the top of the inverted V structure (Figure 1C and D).

The structural arrangement of Thp3^186–470^–Csn12–Sem1 complex resembles Sac3^253–551^–Thp1–Sem1 complex (23). Structural superposition of Thp3^186–470^–Csn12–Sem1 and Sac3^253–551^–Thp1–Sem1 complexes exhibits an overall RMSD of 5.8 Å (Supplementary Figure S5A), indicating significant structural differences between these two complexes. Subsequent work showed that Sac3 TPR-like repeats extend further from residue 253 to residue 137 and residues 90–125 form a novel loop to interact with the N-terminal region of Thp1 in Sac3^60–550^–Thp1–Sem1 complex (37) (Supplementary Figure S5B). To examine if the N-terminal residues of Thp3 might adopt a similar conformation and interact with Csn12, we examined the interaction between N-terminal region of Thp3 (residue 1–186) and Csn12 utilizing biochemical experiment. We were unable to detect any interaction of Csn12 with N-terminal region of Thp3 using pull down assay (Supplementary Figure S6). Although we cannot totally exclude a possible weak interaction beyond the detection limit of our assay, Thp3 and Sac3^81–556^ have different structures in their respective N-terminal regions based on the structural model predicted by AlphaFold (Supplementary Figure S7A and B).

### The detailed Csn12–Sem1 interactions

**Figure 2.**
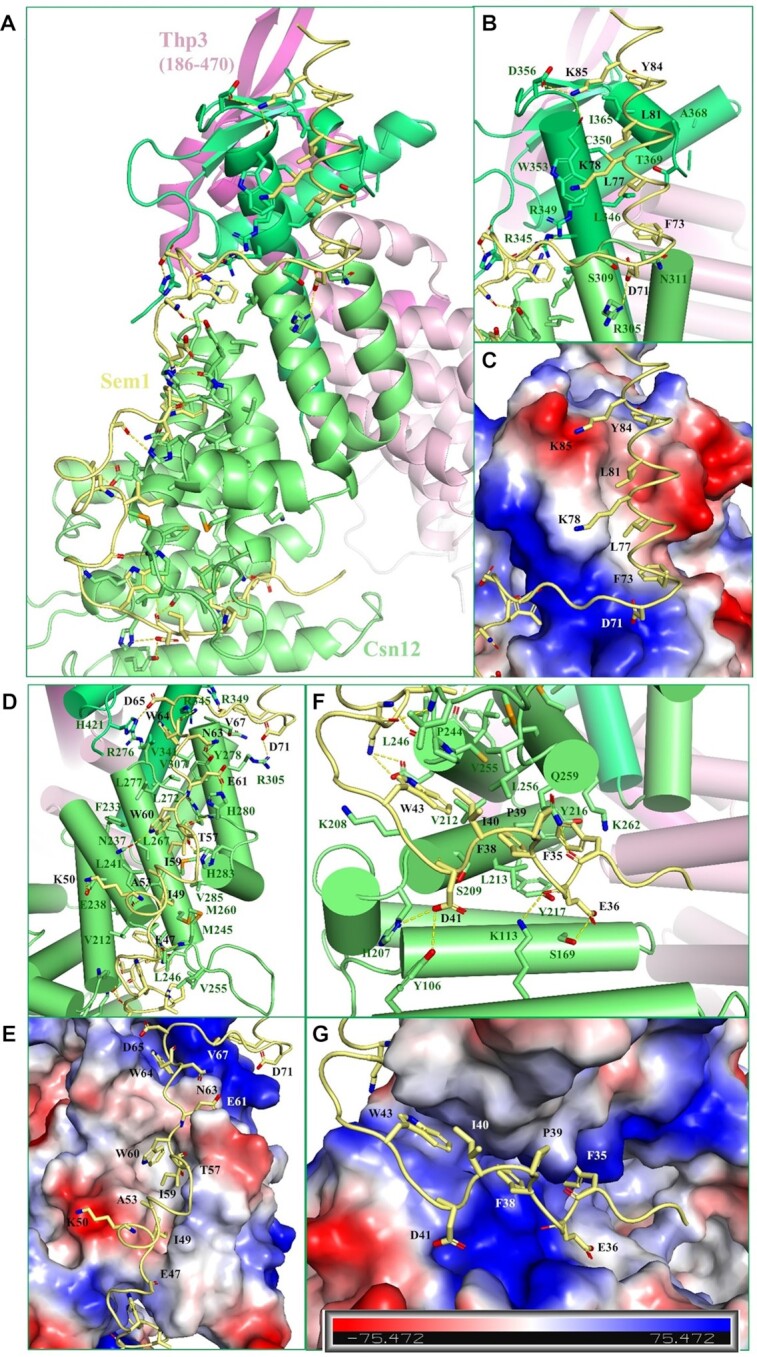
Detailed interactions at the interface between Csn12 and Sem1. (**A**) The overall view of Csn12 and Sem1 interactions. Csn12 and Sem1 are shown in cartoon representation with key interacting residues shown in stick models and colored same as in Figure 1. (**B**) Details of Sem1-Csn12 interactions in the C-terminal helix region of Sem1 (top). (**C**) The same view as in panel (B) with the region on Csn12 shown in electrostatic surface representation. (**D**) Details of Sem1-Csn12 interactions in the middle part of Sem1. The view is rotated 60° along the vertical axis relative to the view in panel (A). (**E**) The same view as in panel (D) with the Csn12 shown in electrostatic surface representation. (**F**) Details of Sem1-Csn12 interactions at the N-terminal region of Sem1 (bottom). The view is rotated 50° along the horizontal axis relative to the view in panel (A). (**G**) The same view as in panel (D) but with the region on Csn12 shown in electrostatic surface representation. The hydrogen bonds and salt bridges are shown as yellow dashed lines. Electrostatic surface potential of Csn12 was calculated in the absence of Sem1 using PyMOL.

In the ternary complex, the fishhook-like Sem1 (32–89) exclusively contacts Csn12 with a larger buried surface area of 2523.3 Å^2^ calculated by PISA server (38). The structure confirmed the biochemical finding that Sem1 stabilizes Csn12 protein exclusively. The Sem1 structure consists of a C-terminal helix serving as the head of the fishhook, and an N-terminal extended loop structure as the body of hook (Figures 1B and 2A). The contacts between Csn12 and Sem1 contain mainly hydrophobic interactions accompanied by hydrogen bonds and electrostatic interactions (Table 2).

**Table 2.** Csn12–Sem1 intermolecular contacts

Csn12 residue	Location	Sem1 residue	Location
**Hydrophobic interactions**			
I365	α16	Y84	α2
C350, I365, A368	α15, α16	L81	α2
W353	α15	K78	α2
R349, W353, T369, L346	α15, α16–α17 loop	L77	α2
S309, N311, L346	α13–α14 loop, α15	F73	α2
L272, R276, Y278, V307, I308, V341,	α11, α11–α12 loop, α13, α15	W64	α1–α2 loop
F233, MSE264, L267, V268, A271, L277, P279	α10, α11, α11–α12 loop	W60	α1–α2 loop
L240, L241,H283, I286	α10, α11–α12 loop, α12	I59	α1–α2 loop
L240, L241, C243, MSE245, MSE260, V285	α10, α10–α11 loop, α11, α12	I49	N-terminal loop of α1
K208, V212, P244, L246, L256	α9, α10–α11 loop	W43	N-terminal loop of α1
L246, V255	α10–α11 loop	I40	N-terminal loop of α1
L246, V255	α10–α11 loop	P39	N-terminal loop of α1
S209, V212, L213, L256, Q259	α9, α10–α11 loop, α11	F38	N-terminal loop of α1
L213, Y216, Y217, Q259, K262	α9, α11	F35	N-terminal loop of α1
**Hydrogen bond interactions**			
W353 O	α15	K85 NZ	α2
R349 NH2	α15	V67 O	α1–α2 loop
R345 NH2	α15	W64 O	α1–α2 loop
Y278 OH	α11–α12 loop	N63 OD1	α1–α2 loop
Y278 O	α11–α12 loop	E61 N	α1–α2 loop
N237 OD1	α10	W60 NE1	α1–α2 loop
H280 N	α11–α12 loop	I59 O	α1–α2 loop
H283 NE2	α11–α12 loop	T57 O	α1–α2 loop
L241 O	α10	K50 N	N-terminal loop of α1
E238 OE1	α10	K50 NZ	N-terminal loop of α1
MSE245 N	α10–α11 loop	E47 O	N-terminal loop of α1
S209 N	α9	D41 O	N-terminal loop of α1
S209 OG	α9	D41 O	N-terminal loop of α1
Y106 OH	α5	D41 OD2	N-terminal loop of α1
K113 NZ	α5	E36 O	N-terminal loop of α1
S169 OG	α7	E36 OE2	N-terminal loop of α1
Q259 NE2		F35 O	N-terminal loop of α1
**Salt bridge interactions**			
D356 OD1	α15–α16 loop	K85 NZ	α2
R305 NH1	α13	D71 OD2	α1–α2 loop
H421 NE2	C-terminal loop of β3	D65 OD2	α1–α2 loop
H280 NE2	α11–α12 loop	E61 OE1	α1–α2 loop
E238 OE2	α10	K50 NZ	N-terminal loop of α1
H207 ND1	η1–α9 loop	D41 OD2	N-terminal loop of α1

The interactions were identified with PISA, and by visual inspection in Coot with donor-acceptance cutoff distance of <3.3 Å for hydrogen bond and ≤3.5 Å for salt bridge.

The C-terminal helix of Sem1 displays amphiphilic feature. The hydrophobic residues (F73, L77, L81 and Y84) are all lined inside and anchor to the nonpolar surface formed by the hydrophobic residues on α15 and α16 helices of Csn12, while the hydrophilic residues on the helix are exposed to the solvent (Figure 2B and C). Additional salt bridges between K85 of Sem1 and D356 of Csn12, D71 of Sem1 and R305 of Csn12 further stabilize the interaction of the C-terminal helix of Sem1 with Csn12 (Figure 2B and C).

The meandering C-shaped loop of Sem1 fits nicely into a large continuous surface groove of Csn12 and is roughly divided into three parts: residue segments 32–44, 45–65 and 66–72 forming the bottom, middle and top parts of the hook, respectively (Figure 2A). The middle part (45–65) is roughly parallel to the C-terminal helix whereas the top (66–72) and the bottom (32-44) parts are roughly perpendicular to the C-terminal helix (Figure 2A). Key hydrophobic residues (F35, F38, I40, W43, I49, I59, W60, W64) from the Sem1 loop fit into the nonpolar groove formed by residues on the surface of helices α9–α13 from Csn12 helical domain (Figure 2D–G), which contribute to a large portion of the buried surface area to stabilize the Csn12 structure. In addition, extensive hydrogen bonds and salt bridges flank the hydrophobic interactions to further stabilize the contacts between Csn12 and Sem1. In the middle region, K50 of Sem1 forms salt bridge with E238 of Csn12. T57, I59, W60, N63 and W64 of Sem1 form hydrogen bonds with H283, H280, N237, Y278 and R345, respectively. E61 of Sem1 forms salt bridge with H280 of Csn12, and D65 of Sem1 forms salt bridge with H421 of Csn12 (Figure 2D & E). In the bottom region, multiple hydrogen bonds and salt bridges are formed between Csn12 and Sem1 (Figure 2F & G). For instance, E36 of Sem1 forms hydrogen bond with S169 of Csn12. D41 mediates two hydrogen bonds with Y106 and S209 of Csn12. The main chain carbonyl of F35 in Sem1 forms a hydrogen bond with the side chain of Q259 of Csn12. Additionally, intramolecular interactions within Sem1 also stabilize Sem1 conformation. A salt bridge is formed between E68 and K78, which orientate F73 and L77 side chains to fit into the nonpolar groove on Csn12. The side chain of N58 forms hydrogen bonds with the main chain carbonyl group of W60 and side chain of E62, so the hydrophobic side chains of I59 and W60 fit into the nonpolar groove on Csn12. Interestingly, while it is almost unstructured in the other Sem1-containing complexes, the non-conserved residues 47–57 of Sem1 adopts a well-defined loop and a short helical conformation when interacting with Csn12 (Figures 1B and 2A).

### Analysis of the Csn12–Thp3 binding interface

**Figure 3.**
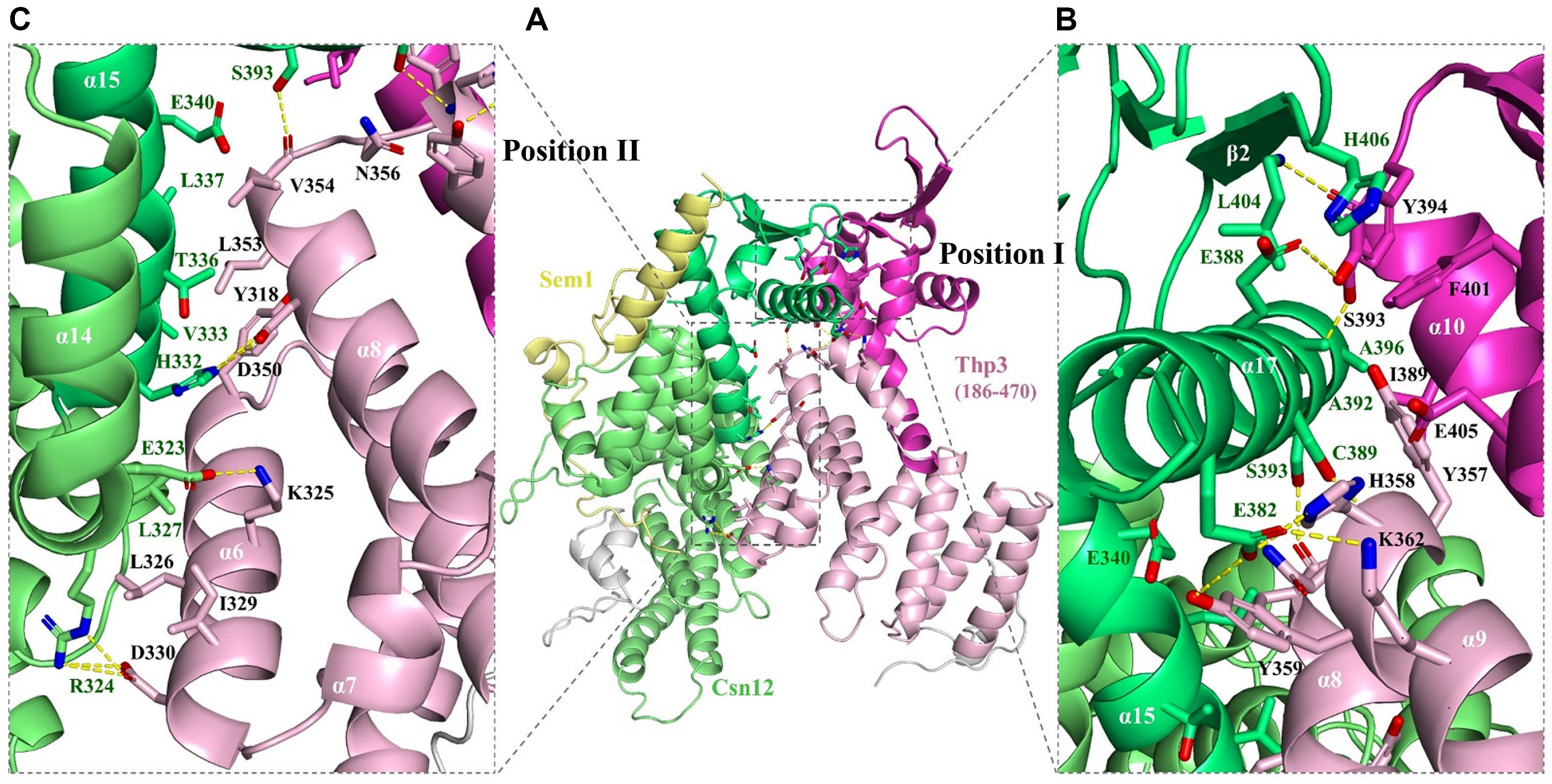
Detailed intermolecular interactions at the Thp3–Csn12 interface. (**A**) The overall structure of Thp3–Csn12–Sem1 complex highlighting the Thp3-Csn12 interface. Thp3 and Csn12 are shown in cartoon representation with key interacting residues shown in stick models and colored same as in Figure 1. (**B**) A close-up view of the interface interactions at position I. The view is rotated 30° along the vertical axis relative to the view in panel (A). (**C**) A close-up view of the interface interactions at position II. The view is rotated 20° along the horizontal axis relative to the view in panel (A). The hydrogen bonds and salt bridges are shown as yellow dashed lines.

Thp3 interacts with Csn12 on the other side of Csn12 molecule, opposite to the Csn12–Sem1 binding interface (Figure 3A). The interface of Thp3 and Csn12 buries a surface area of 1167.1 Å^2^, about half of the total buried surface area at the Csn12-Sem1 interface. The interface between Csn12 and Thp3 is mainly governed by polar interactions including hydrogen bonds and salt bridges, supplemented by hydrophobic interactions (Table 3). The binding interface can be divided into two parts: positions I and II (Figure 3B and C).

**Table 3.** Csn12–Thp3 intermolecular contacts

Csn12 residue	Location	Thp3 residue	Location
**Hydrophobic interactions**			
R324	α14	I329	α6
L327	α14	L326, K325	α6
V333	α15	Y318, L353	α6, α8
T336	α15	L353, V354	α8
L337	α15	L353	α8
E340	α15	V354	α8
A392	α17	I389, Y394	α10, α10–α11 loop
A396	α17	I389, K392	α10
L404	β2	S393, Y394	α10, α10–α11 loop
**Hydrogen bond interactions**			
R324 NE	α14	D330 OD1	α6
R324 NH2	α14	D330 OD1	α6
H332 NE2	α15	D350 OD1	α8
S393 OG	α17	V354 O	α8
E382 OE2	α17	H358 NE2	α9
E382 OE2	α17	Y359 OH	α9
E388 OE1	α17	Y394 OH	α10–α11 loop
C389 SG	α17	H358 N	α9
A392 O	α17	S393 OG	α10
L404 N	β2	S393 O	α10
**Salt bridge interactions**			
E323 OE2	α14	K325 NZ	α6
R324 NH2	α14	D330 OD2	α6
H332 NE2	α15	D350 OD2	α6
E382 OE1	α17	K362 NZ	α9

The interactions were identified with PISA, and by visual inspection in Coot with donor-acceptance cutoff distance of <3.3 Å for hydrogen bond and ≤3.5 Å for salt bridge.

In position I interface, helix α17 of Csn12 packs against the surface formed by helices α8, α9 and α10 of Thp3 and their corresponding connecting loops. Residue E382 at the N-terminus of α17 helix of Csn12 forms two hydrogen bonds with H358 and Y359 and a salt bridge with K362 on α9 helix of Thp3, which lock α17 in place. Towards the C-terminal portion of α17 helix, Csn12 interacts with Thp3 through four pairs of hydrogen bond (E388 of Csn12 with Y394 of Thp3, C389 of Csn12 with H358 of Thp3, A392 of Csn12 with S393 of Thp3, and S393 of Csn12 with V354 of Thp3). In addition, a main chain hydrogen bond between L404 on β2 of Csn12 and S393 of Thp3 brings two three-stranded β sheets together to form a continuous six-stranded β sheet in their joined WH domains.

Helices α14 and α15 from Csn12 pack against helices α6 and α8 from Thp3, which form the binding interface at postion II (Figure 3C). Residues E323 and R324 on helix α14 of Csn12 form electrostatic interactions with the corresponding K325 and D330 residues on helix α6 of Thp3. H332, located on helix α15 of Csn12, forms a hydrogen bond with D350 on helix α8 of Thp3. Additionally, hydrophobic interactions between Csn12 and Thp3 further strengthen the polar interactions at the position II interface (Figure 3C). The hydrophobic residue L327 on α14 of Csn12 interacts with L326 and I329 residues on α6 of Thp3. The hydrophobic residue V333 on α15 of Csn12 interacts with Y318 on α6 and L353 on α8 of Thp3. L337 on α15 of Csn12 interacts with L353 and V354 on α8 of Thp3.

### Function of Thp3–Csn12–Sem1 complex in nucleic acid binding and mRNA splicing

**Figure 4.**
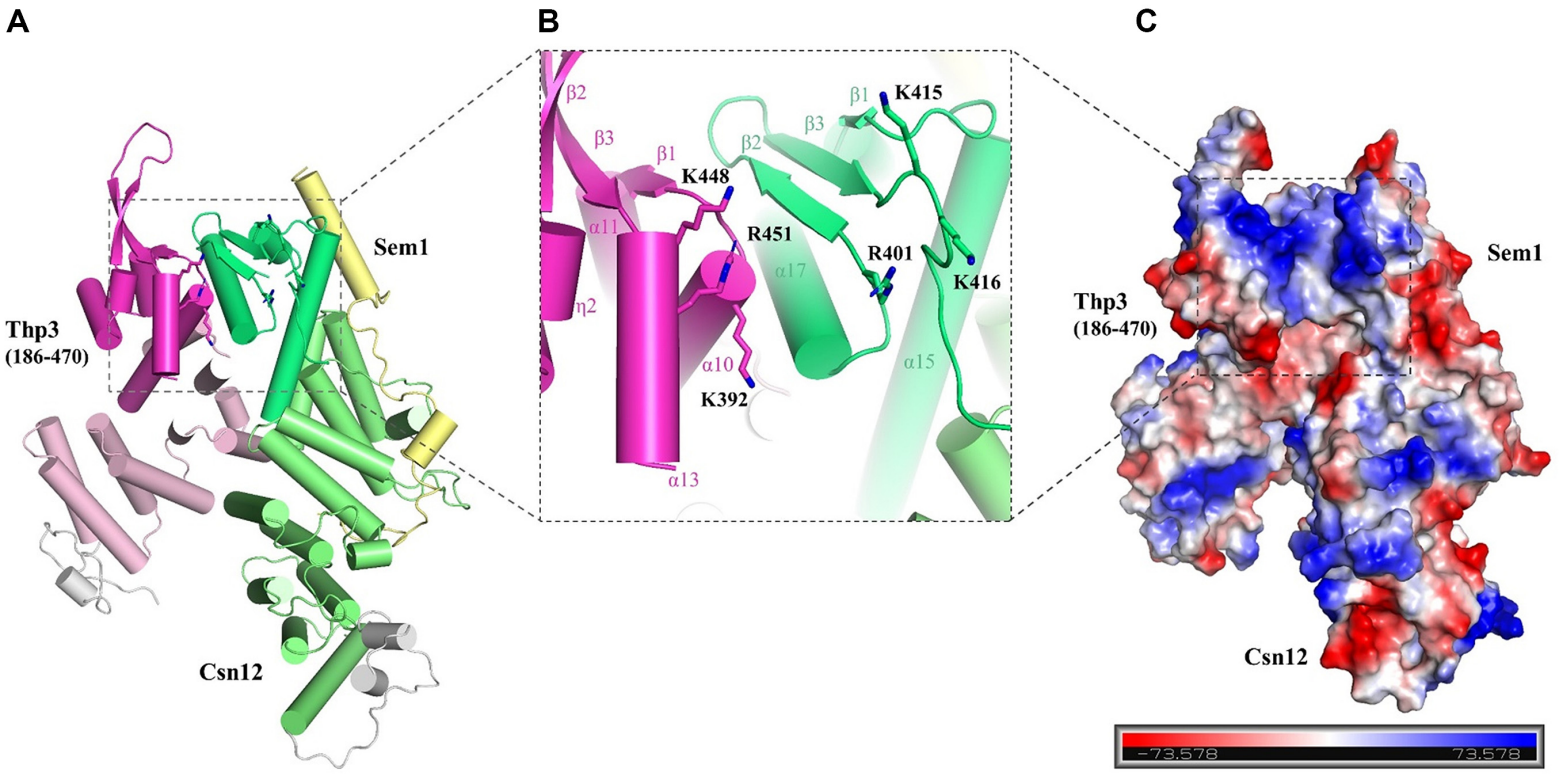
Key basic residues involved in nucleic acid binding in the Winged Helix domains of the Thp3–Csn12–Sem1 complex. (**A**) The overall structure is shown in cartoon representation with the putative nucleic acid binding region boxed (left). (**B**) A close-up view of nucleic acid binding region with key positively charged residues shown in stick models (middle). K462 close to the C-terminus of α13 helix is not shown due to disordering. (**C**) Surface representation with electrostatic potential shown in the same orientation as (A) (right). Electrostatic potential was calculated using PyMOL with negative and positive potentials colored in red and blue. The putative nucleic acid binding region has overall positively charged surface (boxed).

Previous research demonstrated that WH domains are well-known structural motif for nucleic acid binding (39). In the Sac3–Thp1–Sem1 complex structure, the juxtaposition of WH domains from Sac3 and Thp1 forms a continuous positively charged surface to bind nucleic acids (23). In the Thp3–Csn12–Sem1 complex, Csn12 and Thp3 each contains a WH domain. Examination of the C-terminal solvent-exposed dimeric surface formed by Csn12 and Thp3 also revealed a cluster of positively charged residues including K392, K448, R451 of Thp3, and R401, K415, K416 of Csn12 (Figure 4A and B). Surface charge calculation showed that these residues form a continuous positively charged groove (Figure 4C).

**Figure 5.**
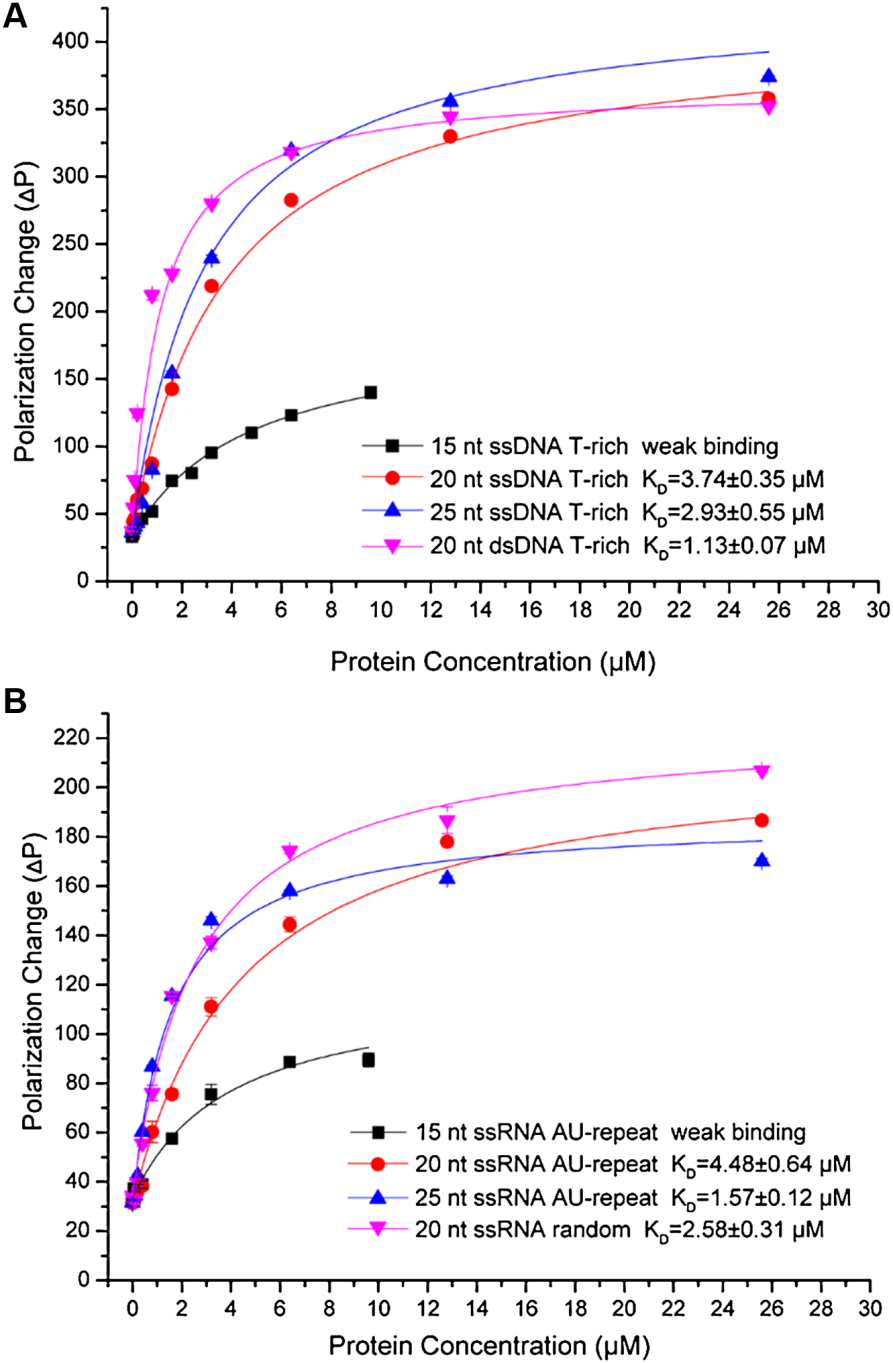
The Thp3–Csn12–Sem1 protein complex has nucleic acid binding activity. (**A**) DNA binding activities measured by fluorescence polarization. 15, 20, 25 nucleotide-long single-stranded (ss) and double-stranded (ds) DNAs with FAM labelled at the 5′ end were used in the binding assay. (**B**) RNA binding activities measured by fluorescence polarization. 15, 20, 25 nucleotide-long ssRNAs with FAM labelled at the 5′ end were used in the binding assay. Each data point represents an average of three independent measurements.

To more systematically assess whether Thp3–Csn12–Sem1 complex has direct nucleic acid binding ability, FP assay was performed utilizing 5′-FAM labeled nucleic acids as probes (Supplementary Table S3). Indeed, we found the Thp3–Csn12–Sem1 complex binds different forms of nucleic acids including double-stranded (ds) DNA, single-stranded (ss) DNA and ssRNA with micromolar affinities (Figure 5A and B). In general, the protein complex exhibits higher binding affinity towards the longer ssDNA. For instance, the protein complex binds 20-nts and 25-nts T-rich ssDNAs with an affinity of 3.7 and 2.9 μM, respectively whereas the binding affinity towards 15-nt ssDNA was too weak to measure (Figure 5A). The complex binds with highest affinity of 1.1 μM to 20-nts dsDNA. More or less similar trend was also observed for ssRNA. The protein complex binds 20-nts and 25-nts AU-repeat ssRNAs with an affinity of 4.5 and 1.6 μM, respectively while it had weaker binding affinity to 15-nts AU-repeat ssRNA (Figure 5B). Both Csn12 and Thp3 proteins contribute to the nucleic acid binding affinity. For instance, Csn12–Sem1 binary complex interacted with 25-nts T-rich ssDNA with an affinity of 33.8 μM, which was more than 11-fold weaker than Csn12–Thp3–Sem1 ternary complex (Supplementary Figure S8).

**Figure 6.**
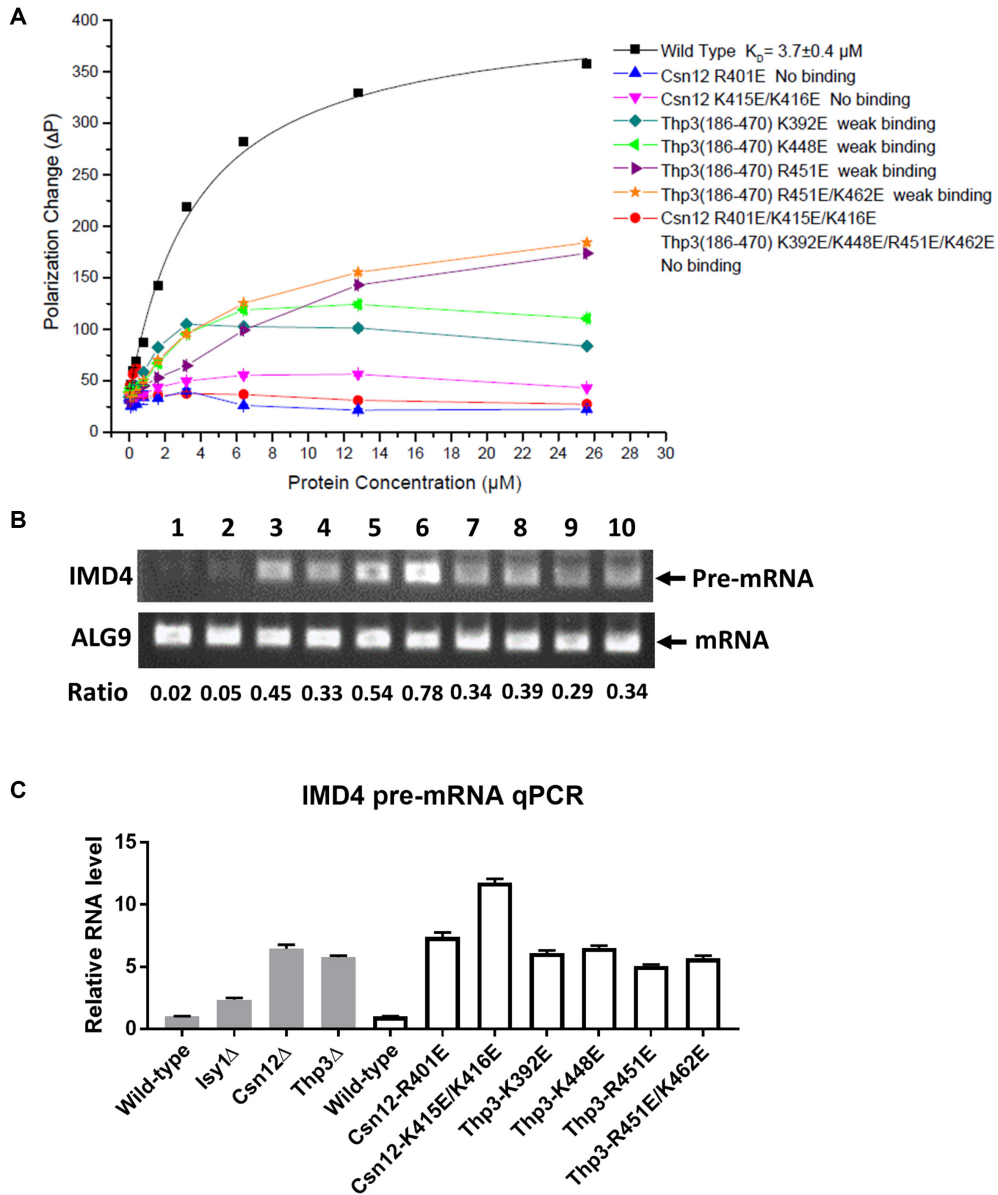
Effects of structure-based mutation of the key basic residues in the Thp3–Csn12–Sem1 complex on nucleic acid binding and mRNA splicing. (**A**) The DNA binding affinity for the Thp3–Csn12–Sem1 complex with different charge mutations was measured by fluorescence polarization assay. 20 nucleotide-long single-stranded (ss) DNA with FAM labelled at the 5′ end was used for the binding assay. Each data point represents an average of three independent measurements. (**B**) The pre-mRNA level of yeast IMD4 gene in three genetic knockouts and different basic residue mutations of Csn12 and Thp3 was examined by endpoint PCR analysis. With an equal amount of cDNAs as template, intron-specific primers of IMD4 gene were amplified by regular PCR for 30 cycles. The intron-less gene ALG9 was amplified similarly and used as a loading control. The PCR-amplified cDNA products were checked on 2% agarose gel. 1. Wild-type, 2. Isy1Δ, 3. Thp3Δ, 4. Csn12Δ, 5. Csn12-R401E, 6. Csn12-K415E/K416E, 7. Thp3-K392E, 8. Thp3-K448E, 9. Thp3-R451E, 10. Thp3-R451E/K462E. (**C**) The pre-mRNA level of yeast IMD4 gene in three genetic knockouts and different basic residue mutations of Csn12 and Thp3 was examined by qPCR assay. The RNA level of IMD4 was normalized to ALG9 internal control. Each data point represents average value (± SD) from three independent measurements.

To investigate the importance of above-mentioned positively charged residues for nucleic acid binding, structure-based charge reversal mutations were introduced in the complex and their effects on nucleic acid binding were assessed by FP assay. Using 20-nts T-rich ssDNA as a probe, all the charge reverse mutations significantly affected nucleic acid binding (Figure 6A). The stronger effect was observed for Csn12 mutations than Thp3 mutations. For example, single residue (Csn12^R401E^) and double residues (Csn12^K415E/K416E^) mutants could completely abolished the nucleic acid binding whereas mutations in Thp3 (Thp3^K392E, K448E, R451E and R451E/K462E^) resulted in the reduced nucleic acid binding (Figure 6A). These results demonstrated that the positively charged surface residues in the WH domains of the Thp3–Csn12–Sem1 complex are critical for nucleic acid binding.

Since the positively charged residues in the WH domains are important for nucleic acid binding, we next accessed their effects on mRNA splicing *in vivo*. CSN12, THP3 or ISY1 genetic knockouts were known to cause splicing defects, e.g. intron retention, with the strongest effect seen for IMD4 gene (13). As a first step, we created genetic knockout of CSN12, THP3 and ISY1 (a positive control) by homologous recombination in *S. cerevisiae* BY4742 strain. Our conventional PCR and qPCR (Figure 6B and C) experiments detected intron retention defects for IMD4 gene i.e. pre-mRNA level was increased by >5-fold for CSN12Δ and Thp3Δ strains as compared to the wild type. Next, site-specific mutations in CSN12 or THP3 were created by homologous recombination similarly as the genetic knockouts. Compared to wild-type cells, the pre-mRNA level of IMD4 gene were all increased for CSN12 (CSN12^R401E, K415E/K416E^) or THP3 (THP3^K392E, K448E, R451E, R451E/K462E^) mutants with the strongest effect seen for CSN12^K415E/K416E^ mutant (more than 10-folds increase), as illustrated in Figure 6B & C. In addition, the ratio of IMD4 pre-mRNA to the total RNA was also increased for CSN12 and THP3 mutants (Supplementary Figure S9A). Intron retention defects for CSN12 and THP3 mutants were also observed for another gene SEC14 (Supplementary Figure S9B). Together, these results demonstrated that the nucleic acid binding site of Thp3–Csn12–Sem1 complex is critical for yeast mRNA splicing *in vivo*.

## DISCUSSION

A large number of machineries, protein complexes are involved in the post-transcriptional gene regulation. Specifically, the Thp3–Csn12–Sem1 protein complex is involved in pre-mRNA splicing. In this study, we reported the first crystal structure of Thp3^186–470^–Csn12–Sem1 complex, which revealed extensive interactions between the three proteins. Csn12 was first described as a subunit of COP9 signalosome complex, which negatively regulates CRLs activity to influence protein ubiquitination and proteasome-mediated protein degradation. The COP9 signalosome complex is comprised of eight protein subunits (CSN1–8). All these proteins are associated through their C-terminal helices to form the helical bundle structure in the COP9 complex (40). Structurally, Csn12 terminates in a WH domain and lacks the C-terminal helix that is important for COP9 complex assembly. Thus, Csn12 is structurally distinct from other subunits of COP9 signalosome complex.

### Determination of the domain boundary of Thp3 for crystallization

Sometimes it is not easy to find the proper domain boundary for protein complex crystallization. For Thp3, we used multiple sequence alignment and secondary structure prediction to find that N-terminal region (residues 1–137) of Thp3 is not conserved and has no regular secondary structures. Based on spontaneous degradation-connected mass spectrometry analysis, we further narrowed down to a stable protein fragment of Thp3 (residues 186–470) to complex with Csn12 and Sem1, which leads to successful crystallization of the ternary complex. Recently, using artificial intelligence, AlphaFold can predict 3D models of proteins far more accurate than before (41). We compared our experimentally determined Thp3 structure with the predicted full length Thp3 model (Supplementary Figure S7B). The model fragment corresponding residues 186–470 are in agreement with the experimental data with an overall RMSD of 1.3 Å. The model also predicted that the N-terminal region (1–185) is highly unstructured except a shorter helix (138–153) (Supplementary Figure S7C). Hence, AlphaFold is a useful modeling tool to find the proper domain boundary for biochemical and structural studies in the future.

### Structural features of Sem1 in different protein complexes

Small versatile protein Sem1 functions as a conformation stabilizer found in many different protein complexes involved in diverse biological processes. Conformational flexibility or intrinsically disordered property allows Sem1 to fit into different surfaces on its partners using different conformations. In the crystal structure of BRCA2–DSS1 complex, DSS1 (Sem1 homolog) is discontinuous and is mainly composed of loops except for a C-terminal short helix (21) (Supplementary Figure S10A). Importantly, DSS1 uses acidic residues to neutralize the positively charged residues on the surface of the OB1 domain of BRCA2. In budding yeast 26S proteasome, Sem1 bridges Rpn3 and Rpn7 in a two-segments binding mode where its C-terminal helix fits into the cleft in Rpn7 and its N-terminal portion binds at the N-terminal helical surface of Rpn3 (42) (Supplementary Figure S10B). Sem1 is also found to interact with different types of ubiquitin by pairing with the positive–hydrophobic–positive patches on the surface of ubiquitin via two acidic–hydrophobic–acidic sequence segments (19). In the Sac3–Thp1–Sem1 complex, yeast Sem1 forms a 19-residue C-terminal helix and an N-terminal discontinuous, extended loop to stabilize Thp1, but also make minor association with Sac3 (23) (Supplementary Figure S10C). Compared with 26S proteasome complex, the binding orientation of Sem1, especially the C-terminal helix is significantly different in the Sac3–Thp1–Sem1 structure. In the Thp3–Csn12–Sem1 complex, Sem1 is visualized as a fishhook-like structure (Figure 2, Supplementary Figure S10D). Unlike other reported complexes where Sem1/Dss1 binds different partners simultaneously, Sem1 only interacts with Csn12 in the Thp3–Csn12–Sem1 complex. The orientation of the C-terminal helix of Sem1 is similar to Sem1 in the Sac3–Thp1–Sem1 complex. The Sem1 fragment involved in Csn12 binding not only includes the highly conserved residues, but also contains a highly divergent region (residue 39–57), which forms a well-defined conformation upon interacting with Csn12, especially residues 51–55 fold into a short helix (Supplementary Figure S10D).

### Nucleic acid binding activity of Thp3–Csn12–Sem1 complex

**Figure 7.**
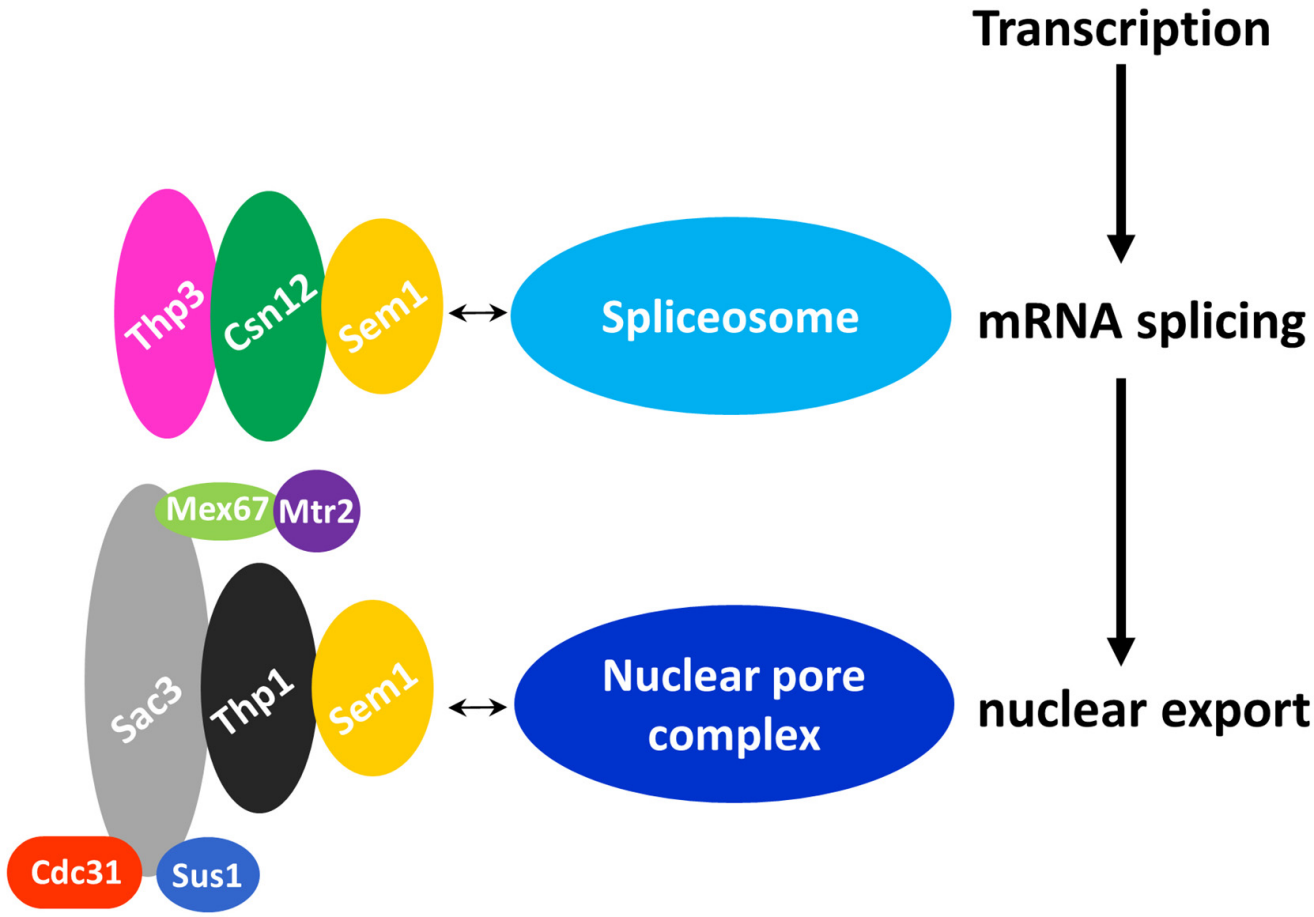
A model of two PCI domain-containing protein complexes in post-transcriptional gene regulation. Thp3–Csn12–Sem1 and Sac3–Thp1–Sem1 complexes have overall similar structural fold and contain the same Sem1 cofactor. Winged helix (WH) domains of Thp3 and Csn12 presumably bind pre-mRNA. Thp3–Csn12–Sem1 complex can also interact with the spliceosome complex through other domains to bring pre-mRNA close to spliceosome for splicing. After splicing, mRNA is pulled to the Sac3–Thp1–Sem1 complex through the higher affinity interaction of the WH domains of Sac3 and Thp1 with mRNA. The Sac3–Thp1–Sem1 complex is part of the TREX-2 complex that also contains Mex67, Mtr2, Sus1 and Cdc31 proteins, which promotes association of mRNA–TREX-2 complex with nuclear pore complex to facilitate mRNA nuclear export.

Winged helix domains are common nucleic acid binding domains. In the Sac3–Thp1–Sem1 complex, the juxtaposition of WH domains of Sac3 and Thp1 generates a positively charged surface for binding nucleic acids (Supplementary Figure S11). The complex can bind to different forms of nucleic acids, most preferably poly(U) RNAs with an optimal length of 25 bases. For instance, the protein complex has a binding affinity of 175 nM for poly(U)_25_ RNA (23). In this study, we found that Thp3–Csn12–Sem1 complex can also bind different nucleic acids. In general, it prefers longer nucleic acids such as 25-base AU-repeats RNA with an affinity of 1.6 μM (Figure 5B). Structure-based mutagenesis confirmed the importance of this positively charged surface in nucleic acid binding and mRNA splicing. If the binding constants are compared, Sac3–Thp1–Sem1 complex exhibits higher nucleic acid binding affinity than Thp3–Csn12–Sem1 complex. Surface charge calculation also showed that the Sac3–Thp1–Sem1 complex has more continuous positively charged surface area than Thp3–Csn12–Sem1 complex (Figure 4C, Supplementary Figure S11C). If we assume WH domains from both complexes serve as the RNA binding platform, our result suggested a sequential binding mechanism for RNA processing (Figure 7). In this proposed sequential model, the pre-mRNA is first captured by Thp3–Csn12–Sem1 complex during transcription to be targeted to spliceosome for mRNA splicing. Following splicing, mRNA can be pulled to the TREX-2 complex due to its higher binding affinity to prepare the processed mRNA for nuclear export. An important question to be addressed in the future is whether WH domains of Thp3–Csn12–Sem1 complex have a preference for intron-containing RNA whereas Sac3–Thp1–Sem1 complex prefers intron-less RNA.

### Connection of the Thp3–Csn12–Sem1 complex to the spliceosome machinery

The spliceosome is a large macromolecular machine consisting of five small nuclear RNP (U1, U2, U4, U5 or U6 snRNPs) and several non-snRNP factors. The snRNPs assemble dynamically on the pre-mRNA together with the non-snRNP protein factors, catalyze the two trans-esterification reactions to produce the mature mRNA by excising the intron(s). Using TAP coupled to mass spectrometry, Csn12 was found to physically interact with SMB1 (SmB), SMX2 (SmG) and SMX3 (SmF) proteins and associate with U1 snRNP, U2 snRNP and Prp19-associated complexes (17). SmB, SmG and SmF are subunits of the ring-shaped heteroheptameric Sm complex including SmB, SmD1, SmD2, SmD3, SmE, SmF and SmG that are part of the spliceosomal U1, U2, U4 and U5 snRNPs. So it is conceivable that the Thp3–Csn12–Sem1 complex is directly connected to spliceosome through physical interaction with SmB, SmF and SmG proteins to be recruited to U1, U2, U4 and U5 snRNPs. The detailed interactions between Thp3–Csn12–Sem1 complex and heteroheptameric Sm protein complex await further structural characterization and will be an exciting area of research in the future. In addition, Csn12 was found to be associated with Prp19-associated complex. Prp19 complex functions more specifically during the catalytic activation of spliceosome by facilitating rearrangements within the spliceosome. Prp19-associated proteins either physically interact with Prp19 or are present in the human 35S U5 snRNP (43). So it is another exciting area of research to elucidate how Thp3–Csn12–Sem1 complex is recruited to the Prp19 complex for catalytic activation of the spliceosome. Based on the preliminary data, we speculated that Thp3–Csn12–Sem1 complex uses WH domains to bind pre-mRNA and the other yet-to-be-identified domains to bind snRNPs, which causes efficient loading of the snRNPs (e.g. U1 and U2 snRNPs) onto the emerging pre-mRNA during transcription elongation (Figure 7). By associating with Prp19 complex, the Thp3–Csn12–Sem1 may also be involved in the catalytic activation of the spliceosome after assembly.

In summary, we reported the first structural study of Thp3–Csn12–Sem1 complex, in which Csn12 is stabilized on one side by the fishhook like Sem1 accessory protein, and on the other side by another PCI domain protein Thp3. The overall domain organization and structural architecture is similar to Sac3–Thp1–Sem1 complex. Importantly, these two PCI domain containing protein complexes are involved in two different but related processes of post-transcriptional regulation. The differential nucleic acid binding affinities may hint an ordered RNA processing mechanism from pre-mRNA splicing to mRNA nuclear export across these two complexes (Figure 7). Despite the similar structural fold, significant structural differences allow the selective interactions of Thp3–Csn12–Sem1 and Sac3–Thp1–Sem1 complexes with spliceosome and nuclear pore complexes, respectively. It is conceivable that the specific structural elements in Thp3–Csn12–Sem1 complex create the unique binding surfaces to interact with the spliceosome complex (U1 snRNP, U2 snRNP and Prp19-associated complexes). Future research is required to elucidate which structural features of Thp3–Csn12–Sem1 complex could facilitate its association with the spliceosome complex to function in pre-mRNA splicing.

## DATA AVAILABILITY

Coordinates of the SeMet substituted and native Thp3–Csn12–Sem1 complexes have been deposited in the Protein Data Bank (PDB) (PDB code: 7EWF, 7EWM).

## Supplementary Material

gkac634_Supplemental_File
