# Supplementary material for: Structural assembly of the nucleic-acid-binding Thp3–Csn12–Sem1 complex functioning in mRNA splicing
Source: Nucleic Acids Res. 2022 Jul 29;50(15):8882–97. doi: 10.1093/nar/gkac634 (PMC9410885; doi:10.1093/nar/gkac634)
Supplement: gkac634_Supplemental_File [file gkac634_supplemental_file.pdf]

Supplementary Information

Structural assembly of the nucleic-acid-binding Thp3-Csn12-Sem1 complex functioning in mRNA splicing

Zhiling Kuang[1, 2], Jiyuan Ke[3 *], Jiong Hong[2], Zhongliang Zhu [1, 2 *], and Liwen Niu [1, 2 *]

[1] Hefei National Laboratory for Physical Sciences at the Microscale, Division of Molecular and Cellular Biophysics, University of Science and Technology of China, Hefei, Anhui 230026, China

[2] School of Life Sciences, University of Science and Technology of China, Hefei, Anhui 230026, China

[3] Institute of Health and Medicine, Hefei Comprehensive National Science Center, Northwest corner of Susong Rd & Guanhai Rd, Hefei, Anhui 230601, China

*Corresponding authors: Jiyuan Ke, jke@ihm.ac.cn; Zhongliang Zhu, zlzhu63@ustc.edu.cn, Tel. (+86) 551-6360-6324; Liwen Niu, lwniu@ustc.edu.cn, Tel. (+86) 551-6360-3046

Supplementary Table 1. Primer sequences for target genes deletion and mutation

Symbol	Forward primer	Reverse primer
CSN12	CCAGCAACGGTTTCTACAACCAATCCAGCTGA AGCTTCGTACGCTGCAGG	CGTTCAATTATTGACTATTTTTCTAGCATAGGC CACTAGTGGATCTGATA
	AATGTCTGGAGAGAGTTACACAGGAACAGAAA GGTCCAGCAACGGTTTCTACAACCAATC	ATTCTTTTATGAACAAACTTTCTTTTTTTTTCT TCGTTCAATTATTGACTATTTTTCTA
	AGGAAAAAAAAAGAAAATTGTATGTAAAGGA ATGTCTGGAGAGAGTTACA	ATACGACCCTATATATATATATATAATATGATAT TCTTTTATGAACAAACTT
THP3	GAGTAGTTGAGTTTCCAATAACATTCAGCTGA AGCTTCGTACGCTGCAGG	CTTAGGCATCCCGTTTCCCTCTTCAGCATAGG CCACTAGTGGATCTGATA
	CCATAGAATACAAGGAGTAGGAACCGTTCGGC AGCGAGTAGTTGAGTTTCCAATAACATT	GAAAAAAAAAAAAAAAAAAAAAACCAAAATC ATATACCTTAGGCATCCCGTTTCCCTCTTCA
	AAATAGAGATTTGGCTGTCTCGTAAAGAAACC ATAGAATACAAGGAGTAG	ATGTGTTGTGAATATGTATATATTTATTTAGA AAAAAAAAAAAAAAAAAAAACCAAAATC
ISY1	GTAATCAGATACCTGTGCATTCAAACAGCTGA AGCTTCGTACGCTGCAGG	TAAATGTTTGAATAATTTTCCATAGGCATAGG CCACTAGTGGATCTGATA
	TGACACACCGTTATATCGCAAGGCGCACCACA TCAGTAATCAGATACCTGTGCATTCAAA	ATGCTTGTGATGGTCATTCGAAATAGTGCCTC TTCTAAATGTTTGAATAATTTTCCATAG
	TTTCAGGATGCATGAAAGAGCAAATAAAAGTG ACACACCGTTATATCGCA	TCTGCAGCTCGGACATGTTTGAAGATTATAAT GCTTGTGATGGTCATTCG
CSN12-R401E/ K415E-K416E	CCAGCAACGGTTTCTACAACCAATCATGGATG TTGATATAGGATGTTAT	CGTTCAATTATTGACTATTTTTCTATCATTTGC TATGGGGAAAGGGCT
	TTTTGCATACGTTGTGACCGATTAGGC	GATTGGTTGTAGAAACCGTTGCTGGAC
	TAGAAAAATAGTCAATAATTGAACGAAGAA	AAGAGAAGCTAGTACGAATATTTCTTGTCGTT
THP3-K392E/ K448E/R451E/ R451E-K462E	GAGTAGTTGAGTTTCCAATAACATTATGCAGA ATCCTTACGGTCACTT	CTTAGGCATCCCGTTTCCCTCTTCATCATTTTT GTCCCTTTATATCA
	GATAAAAATCAAATTGTGTAGGATATGTT	AATGTTATTGGAAACTCAACTACTCGCT
	TGAAGAGGGAAACGGGATGCCTAAG	TATGTGAAATGTCTGCTTTATGGGAACT

Supplementary Table 2. Gene-specific primer sequences for qPCR

**Symbol**	**Forward primer**	**Reverse primer**
IMD4_I	TTCCAAGGTGTGAAAAAGATCTAAGATG	AATTAATCACACATAATGGGCTTTCC
IMD4_T	CACCAGGTGAATACTTCTACAAGGATG	TTCTTGATGGAACCTTTGTCTACGA
ALG9	CACGGATAGTGGCTTTGGTGAACAATTAC	TATGATTATCTGGCAGCAGGAAAGAACTTGGG
SEC14_I	GTATGTTGTTGCTTTTATTTACTTTTTCTT	CTATTTAAAGTGAAGTCATAGTAATACCACTTG

IMD4_I represents pre-mRNA amplicon using intron-specific primer set and IMD4_T represents total mRNA amplicon using exon-specific primer set.

Supplementary Table 3. The oligonucleotide probes used for fluorescence polarization assay

Probe	Types	Length	Labels	Sequences
DNA	ssDNA T-rich	15 bases	5’ FAM	5’-TTTTATTTATTATTT-3’
	ssDNA T-rich	20 bases	5’ FAM	5’-TTTTATTTTATTTTTATTTT-3’
	ssDNA T-rich	25 bases	5’ FAM	5’-TTTTATTTTATTTTTATTTTATTTT-3’
	dsDNA T-rich	20 base pairs	5’ FAM	5’-TTTTATTTTATTTTTATTTT-3’<br>5’-AAAATAAAAATAAAATAAAA-3’
RNA	ssRNA AU-repeat	15 bases	5’ FAM	5’-AUAUAUAUAUAUAUA-3’
	ssRNA AU-repeat	20 bases	5’ FAM	5’-AUAUAUAUAUAUAUAUAUAU-3’
	ssRNA AU-repeat	25 bases	5’ FAM	5’-AUAUAUAUAUAUAUAUAUAUAUAUA-3’
	ssRNA random	20 bases	5’ FAM	5’-CAGCUAAUGCUUCAGUAGUA-3’

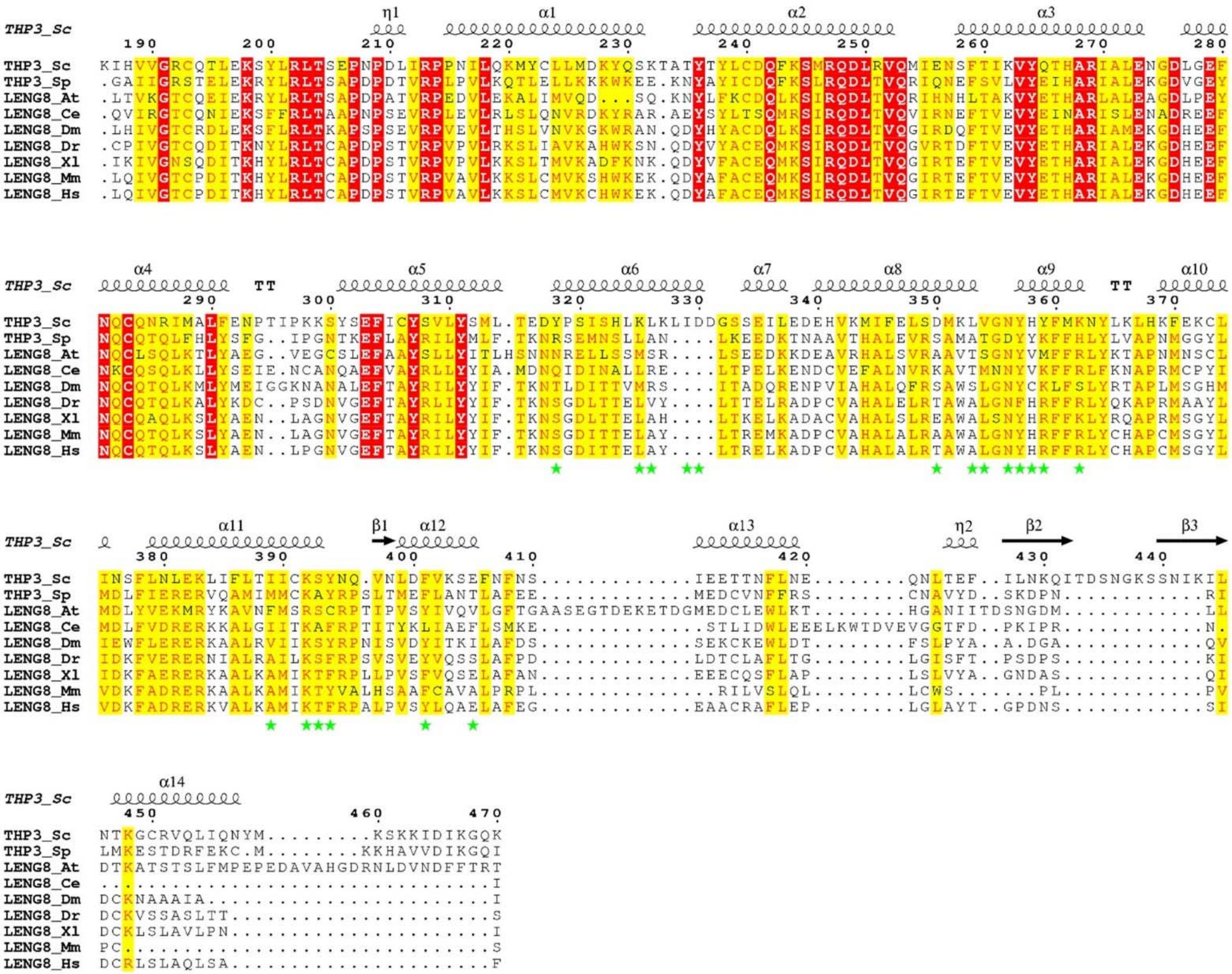

Figure S1. Structure-based multiple sequence alignment of Thp3 and its homolog Leng8. The amino acid sequences corresponding to *S.cerevisiae* Thp3 C-terminal structural region was aligned with the corresponding sequence region of Leng8 from *S.pombe*, *A.thaliana*, *C.elegans*, *D.rerio*, *X.laevis*, *M.musculus* and *H.sapiens* using the software T-coffee. Secondary structure elements of Thp3 are shown on top of the sequences using the ESPript server. The identical residues are colored in white on a red background, whereas similar residues are colored in red on a yellow background. The residues involved in Csn12 heterodimerization are denoted with green asterisks below the alignment.

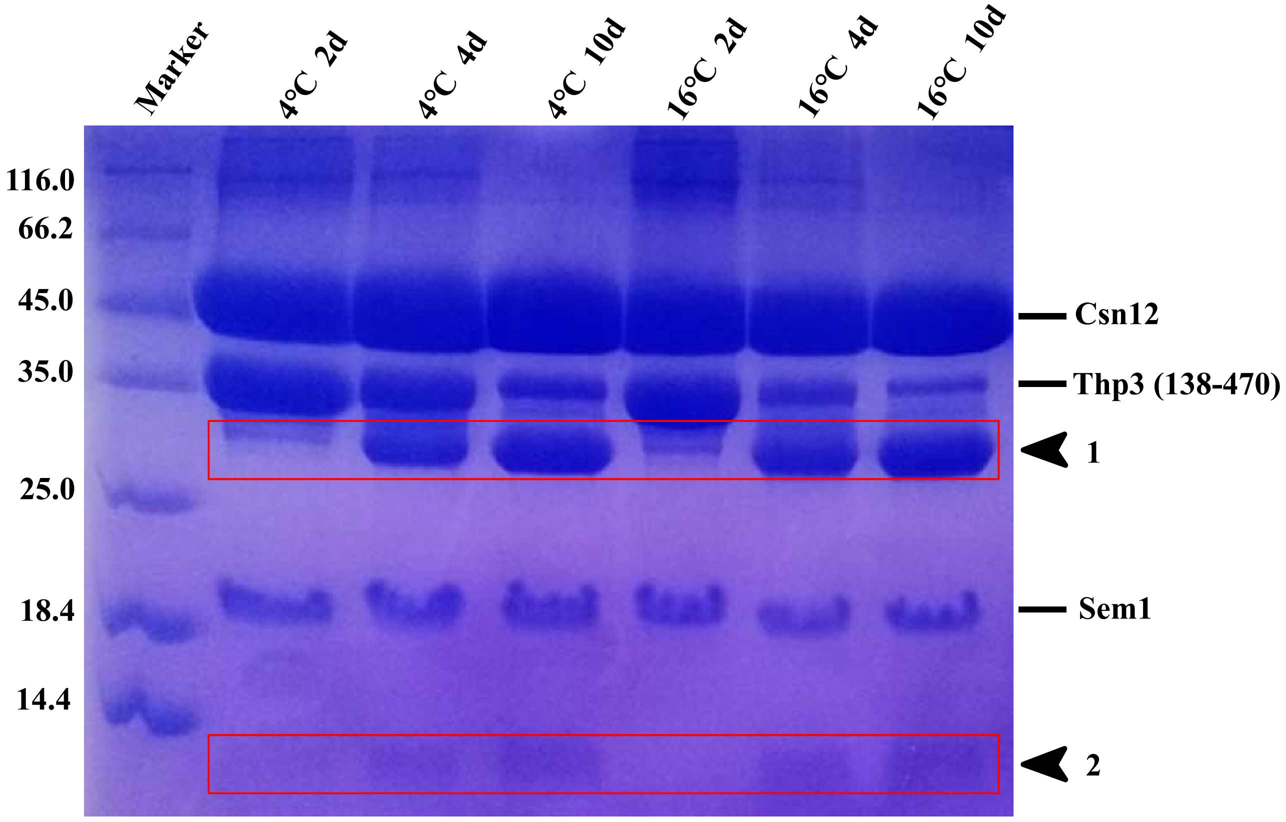

Figure S2. SDS-PAGE of spontaneous degradation products of Thp3(138-470)-Csn12-Sem1 complex. Two stable degradation products of Thp3(138-470) are boxed in red and labeled as product 1 and 2 on the right. Molecular weights in kDa are labeled on the left of the protein markers.

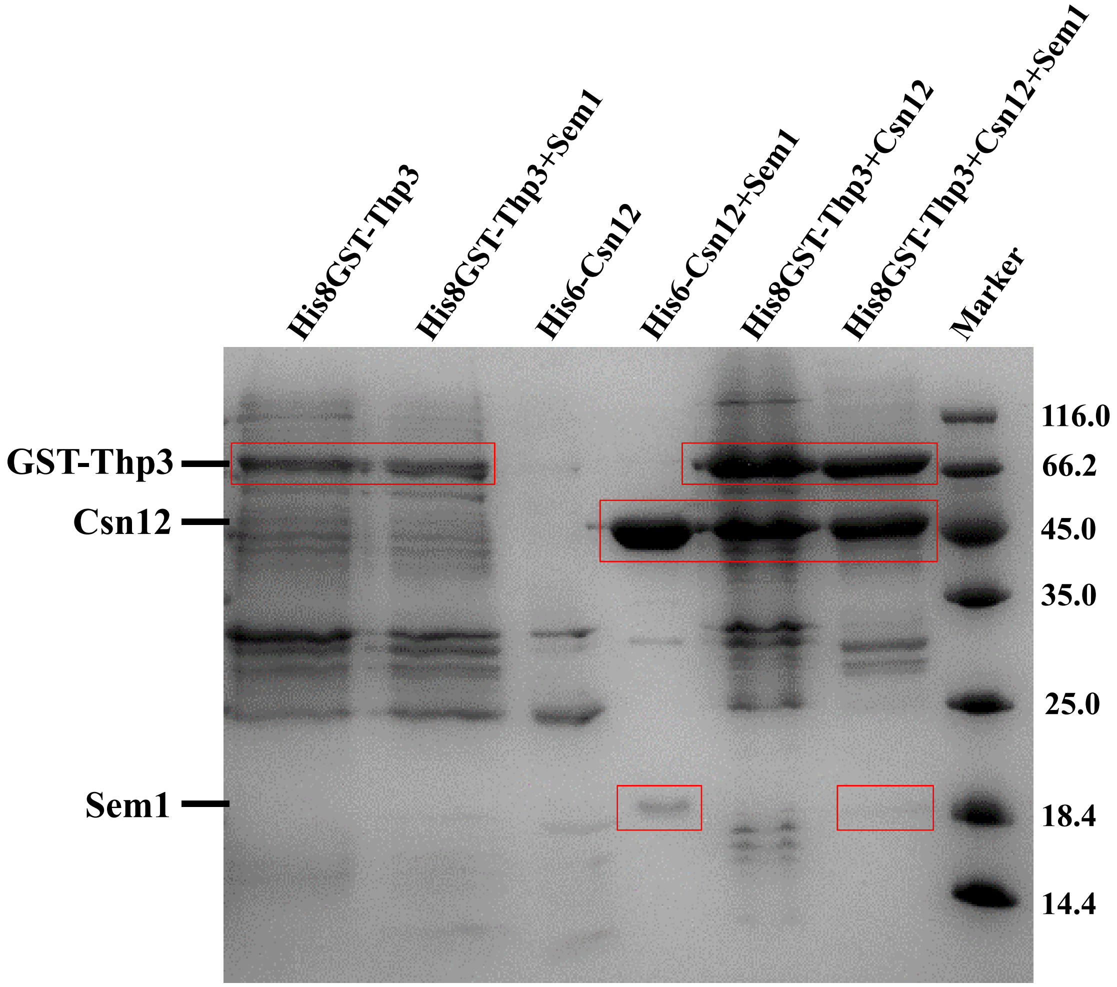

Figure S3. Pull down experiment examining Thp3, Csn12 and Sem1 interactions. His8GST-Thp3 was expressed in the absence and presence of Sem1 protein. His6-Csn12 was expressed in the absence and presence of Sem1. His8GST-Thp3 was coexpressed with Csn12 or Csn12 and Sem1 proteins. Ni beads were used to pull down His tagged protein and its interacting proteins. Bound proteins were released by 1xSDS buffer and analyzed by SDS-gel. The bands corresponding to Thp3-GST, Csn12 and Sem1 are boxed and labeled on the left side of the gel. Molecular weight markers in kDa are labeled on the right side of the gel.

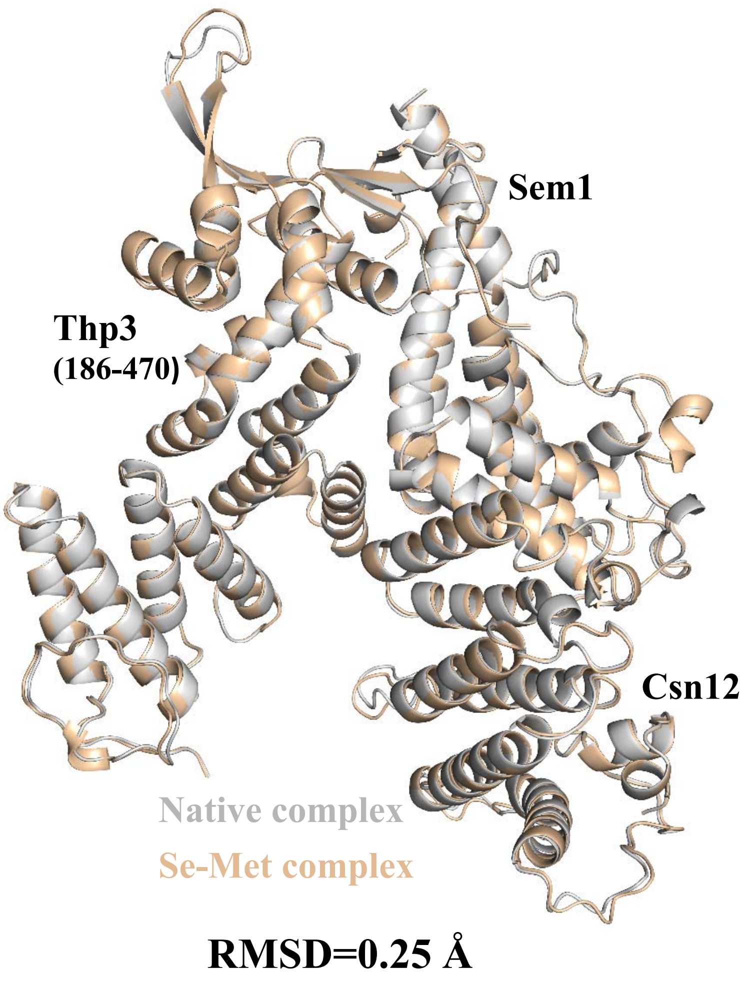

Figure S4. Structural comparison between the native and SeMet substituted Thp3-Csn12-Sem1 complexes. Both native and SeMet substituted Thp3-Csn12-Sem1 structures are shown in cartoon representation. The SeMet substituted structure is colored in orange whereas the native complex structure is colored in gray. The overall root mean square deviation (RMSD) between two structures is 0.25 Å.

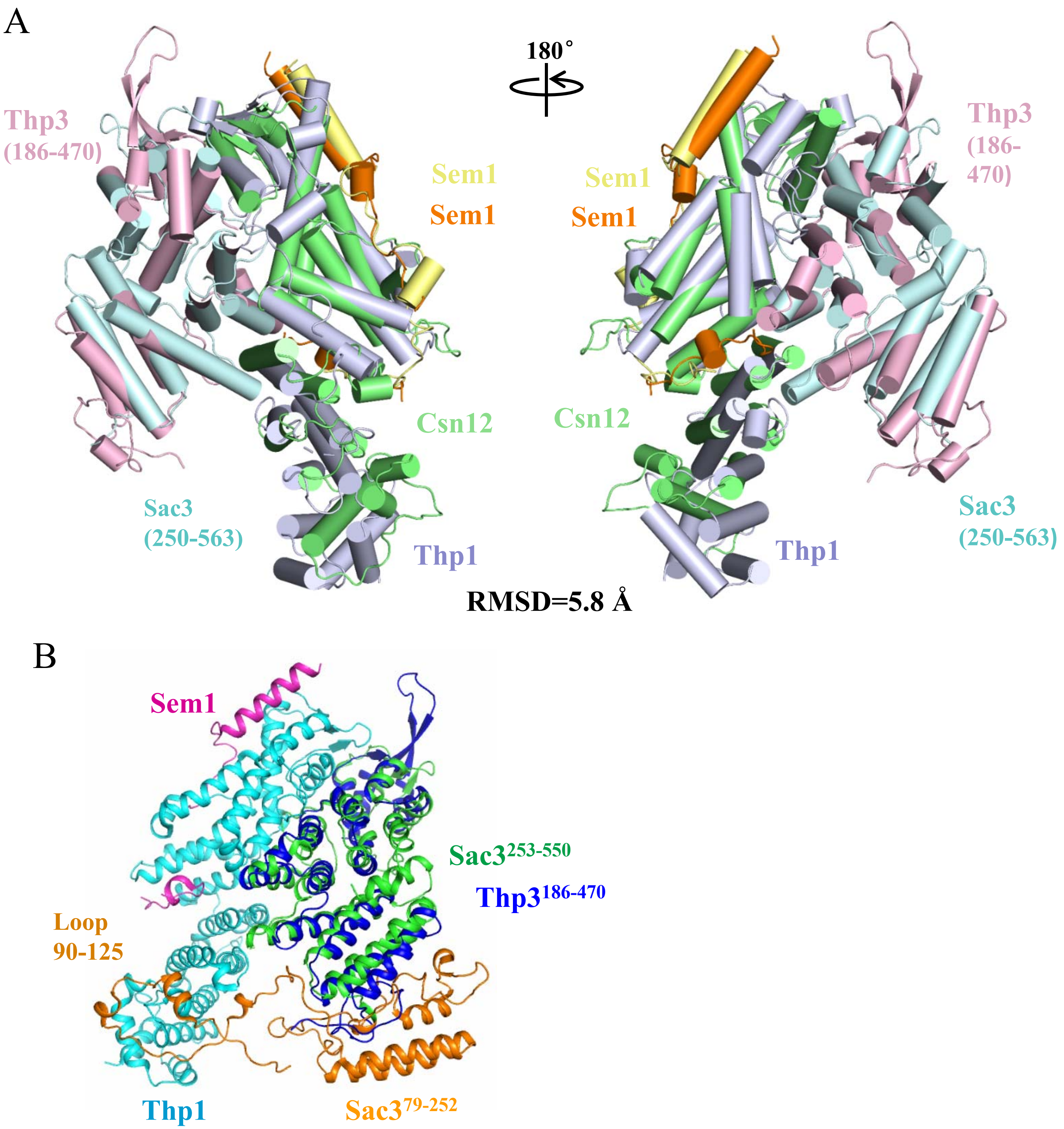

Figure S5. Structural comparison between the Thp3-Csn12-Sem1 and Sac3-Thp1-Sem1 complexes. (**A**) The Thp3-Csn12-Sem1 structure and Sac3$^{250-563}$-Thp1-Sem1 structure (PDB code: 3T5V) are shown in cartoon representation. Thp3, Csn12 and Sem1 structures are colored in pink, green and yellow, respectively whereas the subunits of Sac3-Thp1-Sem1 complex structure are colored in pale cyan, light blue and orange, respectively. The overall root mean square deviation (RMSD) between two structures is 5.8 Å. (**B**) Superposition of Thp3$^{186-470}$ structure with Sac3$^{60-550}$ structure. Sac3$^{60-550}$ structure is shown in the context of the Sac3$^{60-550}$-Thp1-Sem1 complex (PDB code: 5UBP). For simplicity, only Thp3$^{186-470}$ structure is shown. Note that the N-terminal region of Sac3 (residue 79-252) forms additional TPR-like repeats (residue 137-252) and a loop (residue 90-125) extending out to interact with Thp1.

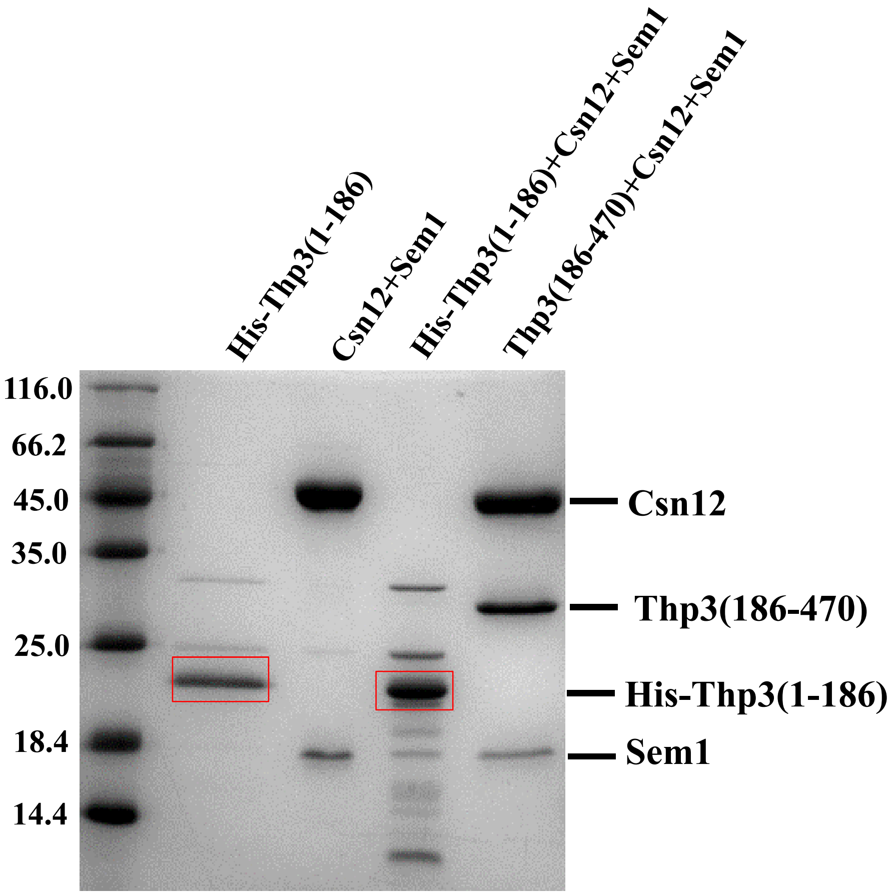

Figure S6. Pull down experiment examining the interaction of N-terminal region of Thp3 (residue 1-186) with Csn12. His tagged Thp3-1-186 was incubated with Csn12-Sem1 complex. Ni beads were used to pull down His tagged protein and its interacting proteins. Bound proteins were released by 1xSDS buffer and analyzed by SDS-gel. The bands corresponding to Thp3, Csn12 and Sem1 are labeled on the right. Molecular weight markers in kDa are labeled on the left side of the gel. His-Thp3-1-186 band is boxed in red. All other bands are either its unstable degradation products or contaminants. No obvious interaction between Csn12-Sem1 and Thp3(1-186) was detected.

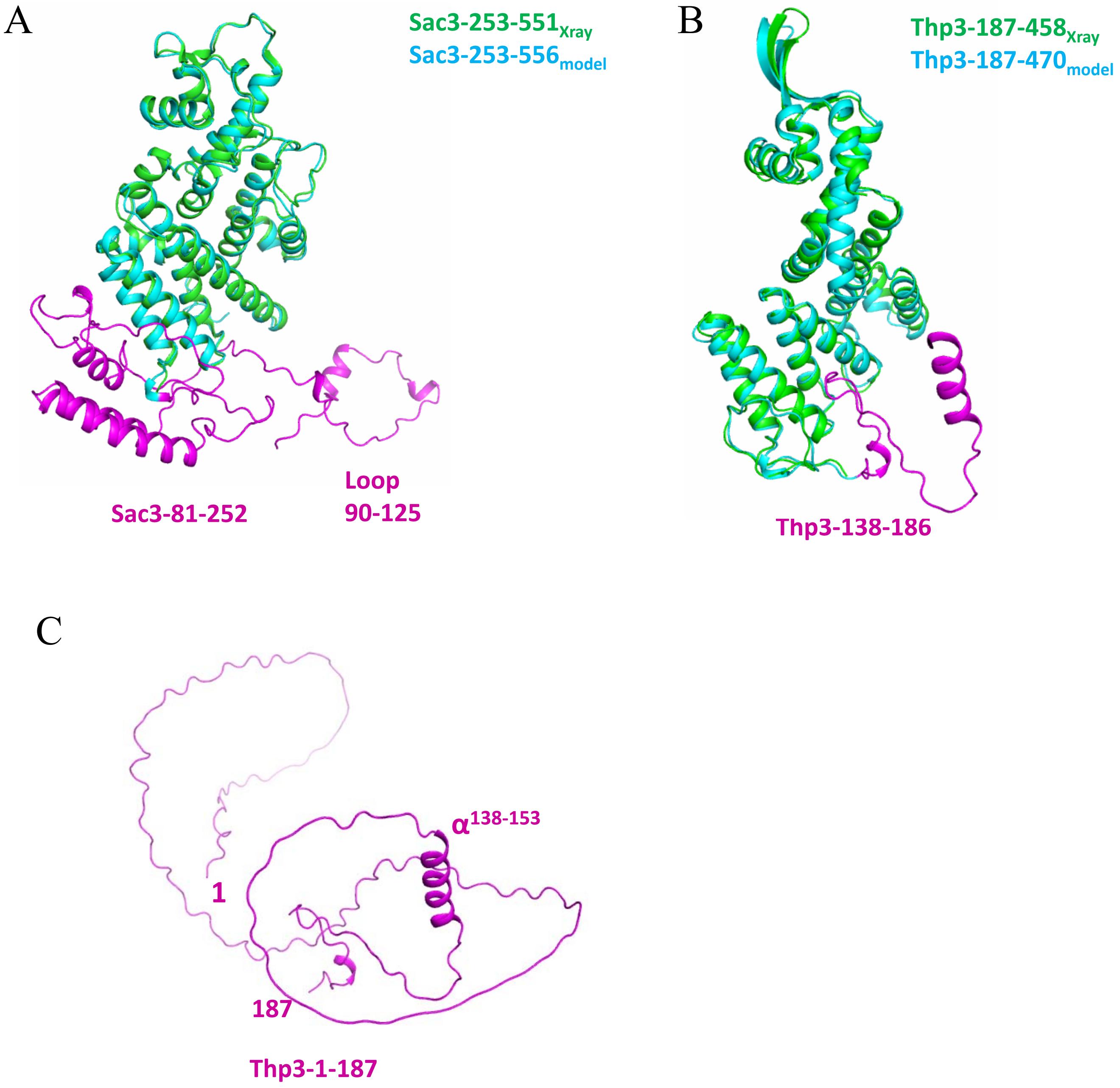

Figure S7. Structural overlay of experimentally determined Sac3$^{253\text{-}551}$ structure with Sac3$^{81\text{-}556}$ model (A) and of experimentally determined Thp3 structure with Thp3 model (B), and the predicted structure for Thp3$^{1\text{-}187}$(C). (**A & B**) The two models are predicted by AlphaFold. All structures are shown in cartoon representation. The experimentally determined Sac3 (residue 253-551) and Thp3 (residues 187-458) are colored in green. The N-terminal fragment (residues 81-252) of the Sac3 model is colored in magenta whereas the C-terminal fragment (residues 253-556) is colored in cyan. Similarly, the N-terminal fragment (residues 138-186) of Thp3 model is colored in magenta whereas the C-terminal fragment (residues 187-470) is colored in cyan. (**C**) Structural model of the N-terminal region of Thp3 (residue 1-187) predicted by AlphaFold. Except for an α helix (residue 138-153), all the other regions are predicted to form flexible loops.

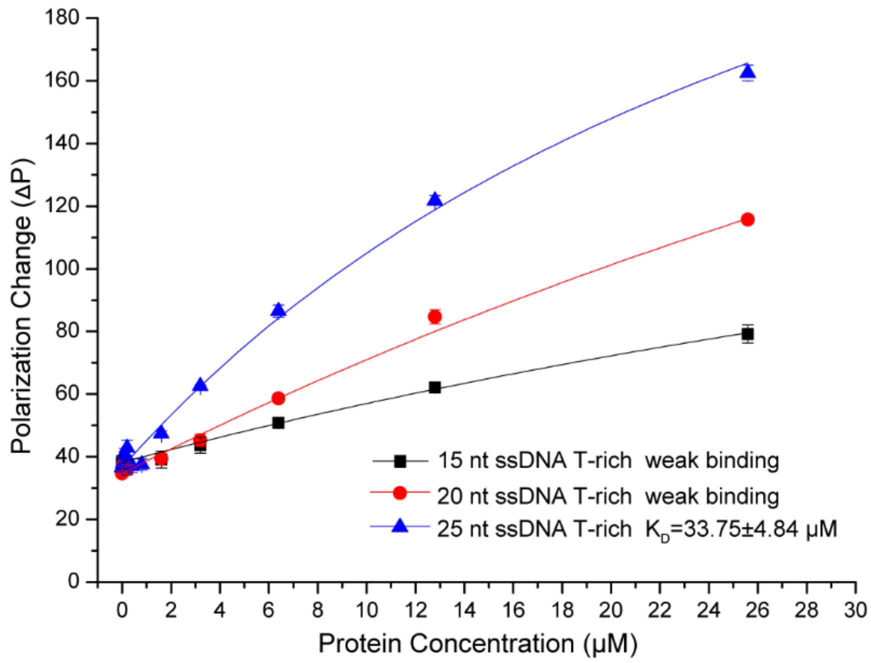

Figure S8. The weak nucleic acid binding activity of the Csn12-Sem1 protein complex. DNA binding activities were measured by fluorescence polarization using 15, 20, 25 nucleotide-long single-stranded (ss) DNAs with FAM labelled at the 5' end. Each data point represents an average of three independent measurements.

A

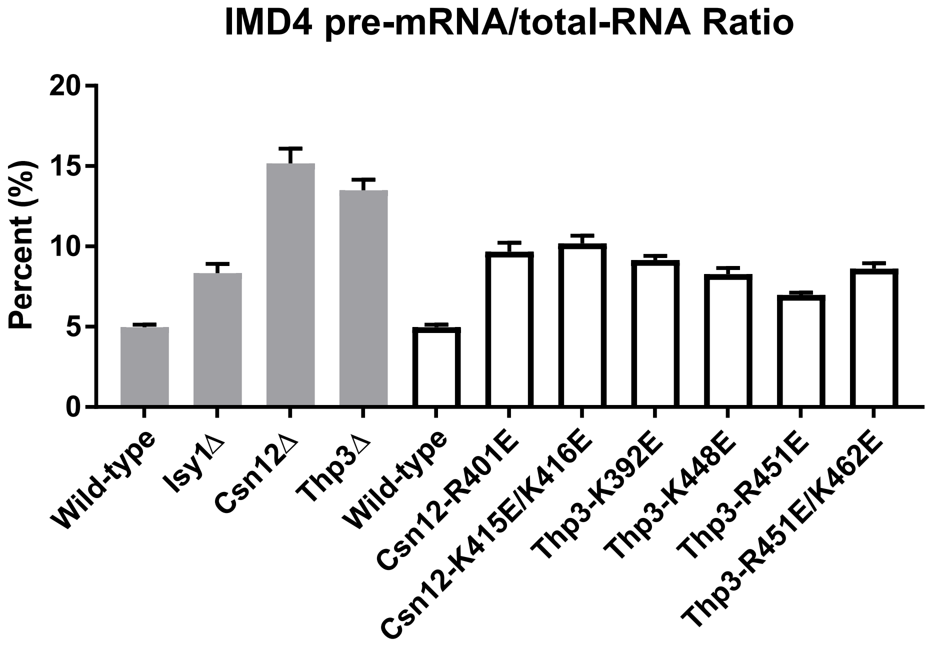

B

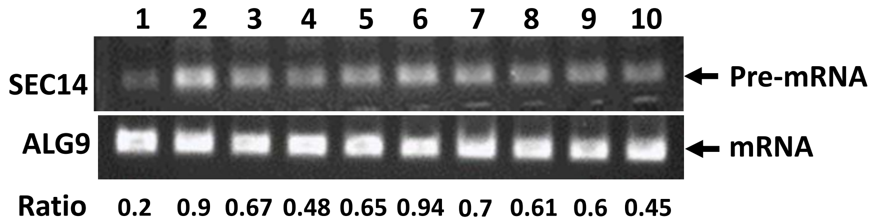

Figure S9. Effects of structure-based mutation of the key basic residues in the WH domains of the Thp3-Csn12-Sem1 complex on mRNA splicing. (**A**) The pre-mRNA level and total mRNA level of yeast IMD4 gene in three genetic knockouts (Isy1Δ, Thp3Δ, Csn12Δ) and different basic residue mutations of Csn12 and Thp3 were measured by qPCR assay using intron-specific primer set (pre-mRNA level) and exon-specific primer set (total mRNA level), respectively. The ratio of pre-mRNA to total RNA indicates the percentage of intron-containing pre-mRNA in total RNA of IMD4 gene. Each datapoint represents average value (± SD) from three independent measurements. (**B**) The pre-mRNA level of yeast SEC14 gene in three genetic knockouts and different basic residue mutations of Csn12 and Thp3 was examined by endpoint PCR analysis. With an equal amount of cDNAs as template, intron-specific primers of SEC14 gene were amplified by regular PCR for 30 cycles. The intron-less gene ALG9 was amplified similarly and used as a loading control. The PCR-amplified cDNA products were checked on 2% agarose gel. 1. Wild-type, 2. Isy1Δ, 3. Thp3Δ, 4. Csn12Δ, 5. Csn12-R401E, 6. Csn12-K415E/K416E, 7. Thp3-K392E, 8. Thp3-K448E, 9. Thp3-R451E, 10. Thp3-R451E/K462E.

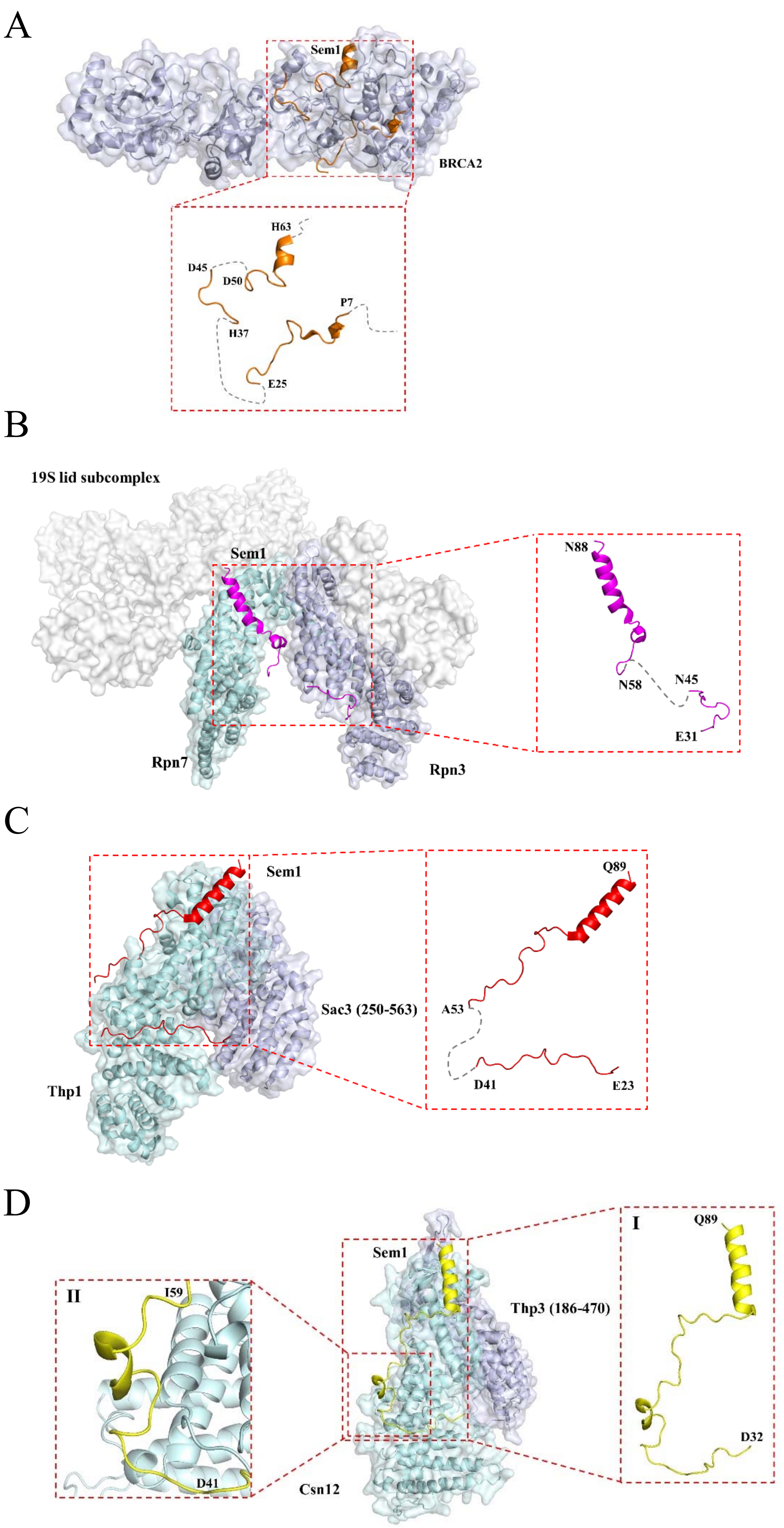
A
Sem1
BRCA2
H63
D45
D50
P7
H37
E25
B
19S lid subcomplex
Sem1
N88
N58
N45
E31
Rpn7
Rpn3
C
Sem1
Q89
Sac3 (250-563)
A53
D41
E23
Thp1
D
I
Q89
Sem1
Thp3 (186-470)
II
I59
D41
Csn12
D32

Figure S10. Comparison of Sem1/Dss1 conformation in different protein complexes. The overall structure is shown as surface and cartoon representations, together with close-up view of Sem1 in cartoon model. Gray dashed lines indicate regions of Sem1/Dss1 that are disordered in the structures. (**A**) Structure of *M. musculus* Dss1 in complex with BRCA2 (PDB ID: 1MIU). Dss1 and BRCA2 are colored in orange and light blue, respectively. (**B**) Structure of *S. cerevisia*e 19S lid subcomplex of 26S proteasome (PDB ID: 3JCK). Sem1 and its interaction partners in complex, Rpn3 and Rpn7 are shown in magenta, light blue and pale cyan, respectively. The other components in 19S lid subcomplex are shown as white surface representation. (**C**) Structure of the Sac3-Thp1-Sem1 subcomplex in *S. cerevisiae* TREX-2 complex (PDB ID: 3T5V). Sem1, Sac3 and Thp1 are shown in red, light blue and pale cyan, respectively. (**D**) Structure of *S. cerevisiae* Thp3(186-470)-Csn12-Sem1 complex. Sem1, Thp3 and Csn12 are colored in yellow, light blue and pale cyan, respectively. A complete Sem1 structure is shown in close-up view I whereas the region with well-defined conformation specific for Csn12 is shown in close-up view II.

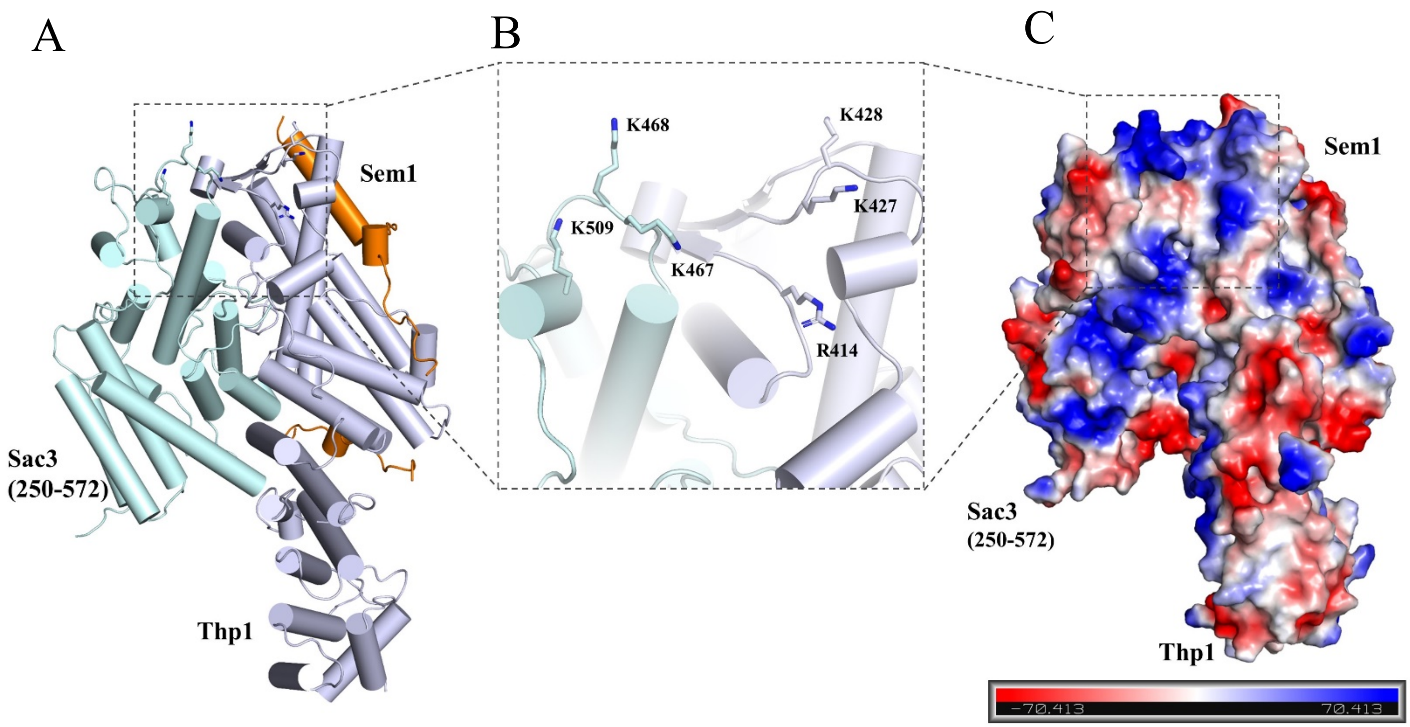

Figure S11. Key basic residues involved in nucleic acid binding in the Winged Helix domains of the Sac3-Thp1-Sem1 complex (PDB ID: 3T5V). (**A**) The overall structure is shown in cartoon representation with the putative nucleic acid binding region boxed (left). (**B**) A close-up view of nucleic acid binding region with key positively charged residues shown in stick models (middle). (**C**) Surface representation with electrostatic potential, shown in the same orientation as (A) (right). Electrostatic potential was calculated using PyMOL with negative and positive potentials colored in red and blue. The putative nucleic acid binding region has overall positively charged surface (boxed).
